# tRNA modifications in cancer: from molecular mechanisms to clinical translation

**DOI:** 10.1186/s40364-026-00919-x

**Published:** 2026-04-11

**Authors:** Cillian H. Cheng, Chi Chun Wong

**Affiliations:** Institute of Digestive Disease and Department of Medicine and Therapeutics, State Key Laboratory of Digestive Disease, Li Ka Shing Institute of Health Sciences, The Chinese University of Hong Kong, Hong Kong SAR, China

**Keywords:** Transfer RNA (tRNA), tRNA modification, tRNA-derived RNAs (tdRs), Molecular mechanisms, Clinical applications, Cancer

## Abstract

tRNA modifications, the most extensively and diversely modified class of RNA across all domains of life, have garnered significant and growing attention in research over the past decade. tRNA modification defects and tRNA fragmentation has been observed within a wide spectrum of cancer types, suggesting their potential as diagnostic and prognostic biomarkers. Mechanistic studies demonstrate that regulatory enzymes for tRNAs and tdRs function as oncogenes or tumor suppressors with vital roles in cancer initiation, progression, metastasis, metabolic rewiring, therapy resistance, and immune evasion, highlighting the therapeutic potential of targeting perturbed tRNA modification machinery in cancer treatment. Herein, we summarize our current understanding of the role of tRNA modifications in cancer, and outline translational and clinical implications for cancer diagnosis and treatment. Emphasis is placed on how tRNA modifications determine the fate of target tRNAs and its influence on protein expression, molecular mechanisms and cell phenotypes. Finally, we discuss the hurdles and potential solutions to translating recent knowledge of tRNA modifications into clinical practice.

## Introduction

Chemical modification is an efficient and highly specific tactic for modulating the function of biological macromolecules, which can occur on proteins, nucleic acids (RNA and DNA), sugars, and lipids [1–5]. More than 170 different types of RNA modifications have been discovered, and recent developments in analytical chemistry, mass spectrometry, and high-throughput sequencing technologies have enabled mapping of the complex landscape of RNA modifications in health and disease states [6–8].

Transfer RNA (tRNA) plays an integral role as an essential adaptor molecule that facilitates the decoding of messenger RNA (mRNA) codons into their corresponding amino acids during protein synthesis in the ribosome based on the genetic code [9, 10]. In 1965, Holley et al. determined the complete chemical structure of a 77 base tRNA^Ala^ from yeast, which included the modified nucleotides inosine (I) and 1-methylinosine (m^1^I) [11]. Since then, a growing body of evidence has indicated that tRNA harbors the most extensive and diverse array of modifications among all classes of RNA species across biological kingdoms, and approximately 80% of the RNA modifications thus far identified are tRNA modifications, with over 100 distinct modifications reported [10, 12–15]. These modifications play important roles in tRNA structure, folding, stability, decoding accuracy, translation efficiency, and overall cellular function, which are primarily contingent upon their location within the tRNA molecule and their chemical properties [16–19]. Besides changes in tRNA function caused by modifications, tRNA abundance is also influenced by a wide array of factors, such as mutations in RNA polymerase III complex genes, transcriptional regulation by oncogenes and tumor suppressors, mutations in tRNA-encoding genes, and alterations in tRNA maturation and splicing (as reviewed elsewhere [20, 21]).

In humans, dysregulation of tRNA modifications is frequently associated with a spectrum of diseases, including in cancer, mitochondrial diseases, neurological disorders, diabetes, and developmental disorders [20, 22–29]. Over the past decade, tRNA modifications have gained prominence in the context of cancer owing to their potential function in dynamically regulating tumorigenesis, metastasis, and therapy responses [12, 30–37]. Aberrant tRNA modifications have been implicated in modulating the translation of oncogenes and tumor suppressor genes, thereby contributing to malignant phenotypes [38, 39]. Specific tRNA modifications are linked to the regulation of tumor metastasis by activating cell adhesion and migration pathways [40–42]. Moreover, dysregulated expression of tRNA-modifying enzymes has been acknowledged as potential biomarkers for cancer prognosis and targets for therapeutic intervention [21, 31, 43].

Recent studies have highlighted the dynamic nature of tRNA biology, revealing that tRNAs are versatile molecules, rather than merely static components of the translation machinery [44–46]. Accumulating evidence suggests that tRNA can be processed in the cytosol into smaller fragments referred to as tRNA-derived RNAs (tdRs), also known as tRNA-derived small RNAs (tsRNAs) or tRNA-derived fragments (tRFs) [47]. These tRNA fragments are not random [48, 49]. Their production is associated with tRNA chemical modifications, resulting in distinct biological functions and regulatory roles in cancer, such as cell viability, cellular differentiation, and the tumor microenvironment (TME) [47, 50–58]. In addition to cytosolic (cyto-)tRNAs, mitochondrial (mt-)tRNAs undergo a range of post-transcriptional modifications by nuclear-encoded tRNA-modifying enzymes generated in cytoplasm and transported into mitochondria to perform their functions [59–62]. These modifications are required for accurately deciphering the genetic code and for stabilizing tRNA [63–65]. The aberrant regulation of mt-tRNA modifications is also frequently linked to the development of cancer [40, 62, 66].

Elucidating the intricate landscape of tRNA modifications and their regulatory enzymes in cancer could pave the way for novel therapeutic opportunities and biomarkers for diagnosis and prognostication [7, 29, 67, 68]. Herein, we reviewed the current understanding of the role of the tRNA modifications in cancer development and explore their potential clinical implications for patients with cancer, with an emphasis on their mechanisms of action, and how they could be therapeutically targeted for cancer prevention or treatment. Additionally, we highlight the many unknown aspects of tRNA modification and propose potential paths forward in this promising area of research.

## The biology and function of tRNA modifications

### Human tRNA gene features and lifecycle of tRNA

In 1956, the existence of tRNA was proposed by Francis Crick to hypothesize that a type of intermediary molecule, containing a tri-nucleotide (triplet) that form base pairs with the genetic template, and would carry a covalently linked amino acid for incorporation into the polypeptide chain [69–71]. Strikingly, the human genome consists of > 610 predicted tRNA genes, producing 432 unique tRNA transcripts from 57 tRNA anticodon families, decoding all 61 amino acid codons for 20 amino acids [72–75]. Approximately half of all the human tRNA genes are transcriptionally silent or poorly expressed, but they appear to have extra-translational roles, acting as insulators preventing heterochromatin repression, facilitating enhancer-mediated transcription activation, causing replication fork pausing, promoting evolutionary recombination events, and contributing to genome organization [75–77]. tRNA genes can be classified as ‘isodecoders’ or ‘isoacceptors’ [78]. tRNA genes that share the same anticodon but have different sequences in the body are termed ‘isodecoders’, and more than 50% of human tRNA genes fall into this category [10, 78, 79]. It is evident that not all isodecoders are expressed in every human cell and their expression is anticipated to vary significantly across different human tissues [80, 81]. The more widely acknowledged term ‘isoacceptors’ refers to tRNA genes that have different anticodons but encode the same amino acid [82]. The degeneracy of the genetic code, meaning that most amino acids are specified by more than one codon, necessitates the existence of isoacceptors [83, 84]. These synonymous codons, recognized by different tRNA isoacceptors, are preferentially used at higher frequencies, resulting in biased codon usage that is observed in nearly all sequenced genomes [84].

**Fig. 1:**
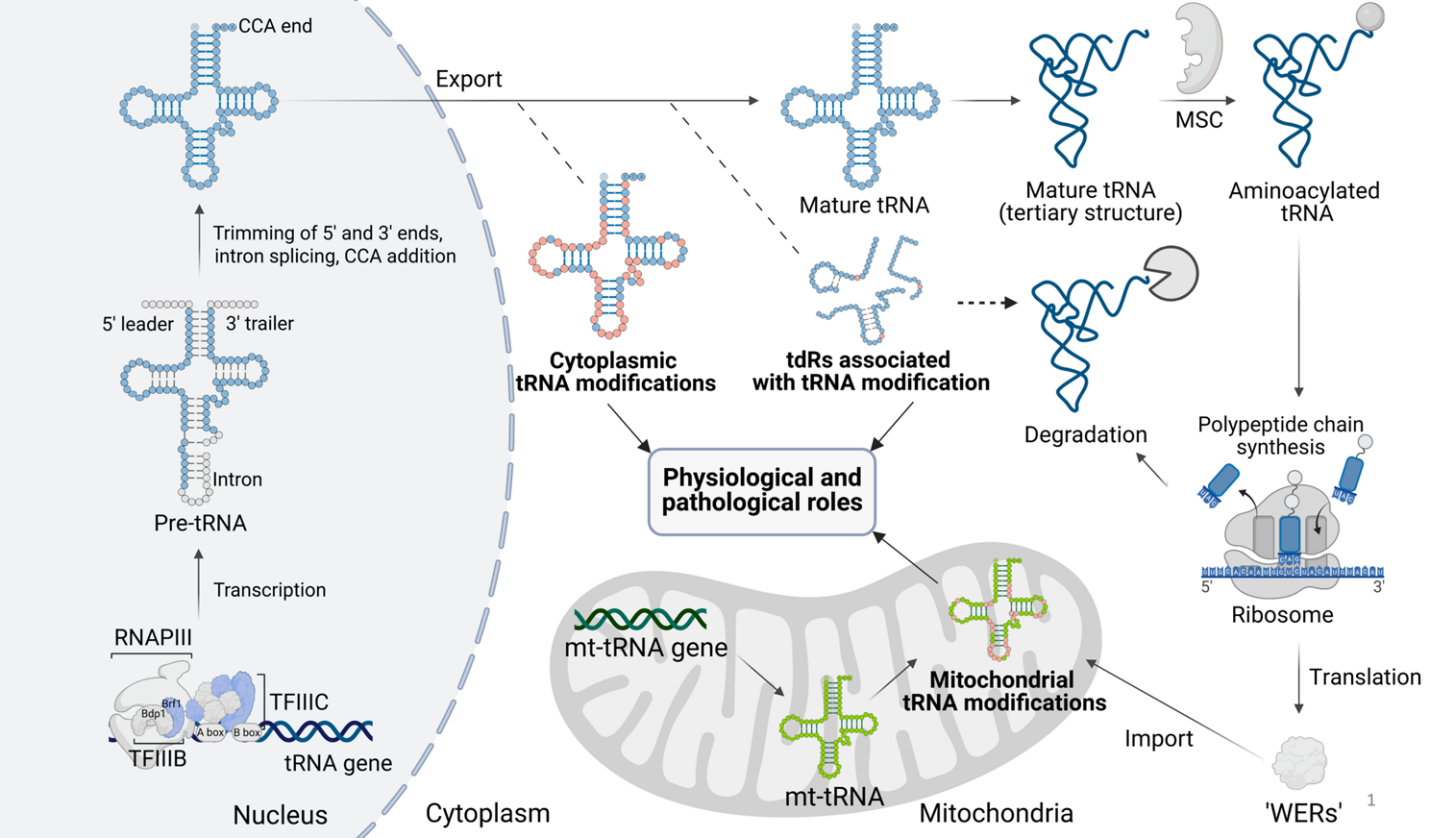
Schematic diagram of transfer RNA (tRNA) life cycle. The lifecycle of a tRNA molecule begins with the transcription of the tRNA gene, a process orchestrated by RNA polymerase III (RNAPIII) in concert with the transcription factors IIIB (TFIIIB) and IIIC (TFIIIC), which collectively facilitate the generation of precursor tRNAs (pre-tRNAs) within the nucleus. Nascent pre-tRNAs subsequently undergo post-transcriptional processing, which includes the trimming of the 5'leader and 3' trailer sequences, intron splicing, and the addition of a 3' terminal trinucleotide (CCA). Following its exit from the nucleus into the cytoplasm, tRNA is processed extensively by a variety of tRNA-modifying enzymes, resulting in a mature and stable form, with specific modifications that shape tRNA-derived RNAs (tdRs) biogenesis in response to environmental stimuli. Next, tRNA folds into a three-dimensional L-shaped structure, while the multi synthetase complex (MSC), composed of aminoacyl-tRNA-synthetases (AARS) and AARS-interacting multifunctional proteins (AIMPs), charges mature tRNAs with their corresponding cognate amino acids. The same charged tRNA can participate in multiple rounds of polypeptide synthesis and translation on ribosomes before being ultimately degraded. The biosynthesis of mitochondrial tRNA (mt-tRNA) entails the transcription of tRNA genes encoded by mitochondrial DNA. Following this, mt-tRNA is modified by mitochondrial tRNA-modifying enzymes, which are synthesized in the cytoplasm and subsequently translocated to the mitochondrial matrix to execute their functional roles. Dysregulation of both cytosolic and mitochondrial tRNA modifications, as well as alterations in the biogenesis and modification status of tdRs, has emerged as a critical driver of various physiological and pathological processes, including cancer. ‘WERs’, ‘writers’, ‘erasers’, and ‘readers’

The lifecycle of a tRNA molecule begins with several processing steps [85, 86]. Transcription of tRNA gene is catalyzed by RNA polymerase III (RNAPIII), which acts in concert with the transcription factor IIIB (TFIIIB) and transcription factor IIIC (TFIIIC) complexes to generate ~100 nucleotide nascent precursor tRNAs (pre-tRNAs) in the nucleus [87]. This process starts with the binding of the six-subunit TFIIIC to the A and B boxes within the internal promoters of tRNA genes, which correspond to D- (dihydrouridine) and T- (thymidine, pseudouridine, and cytidine-containing or TΨC) loops of mature tRNAs [88]. The TFIIIB complex, which recognizes upstream regions of tRNA genes and directs RNAPIII binding, is recruited through the interaction of one of the TFIIIC subunits with two subunits of TFIIIB, Brf1 and Bdp1 [89]. An initial step in pre-tRNA maturation involves the removal of the 5'leader and 3' trailer sequences by endoribonucleases ribonuclease (RNase) P and RNase Z, respectively, while the short intron is excised by the tRNA-splicing endonuclease complex [90, 91]. Next, tRNA nucleotide transferase adds the CCA sequence to the 3' end, and the tRNA exits the nucleus into the cytoplasm, where it undergoes further processing by various tRNA-modifying enzymes to become mature and stable [92]. Mature tRNA experiences coaxial stacking, particularly between the D- and T-loops, which creates the distinctive L-shaped tertiary structure [93]. Hypomodified or defective mature tRNAs/pre-tRNAs either undergo fragmentation or are degraded through a system known as ‘rapid tRNA decay’ (RTD) [94, 95]. Afterwards, the multi-synthetase complex (MSC), composed of aminoacyl-tRNA synthetases (aaRSs) and aaRS-interacting multifunctional proteins (AIMPs), charges the mature tRNAs with their corresponding amino acids [96]. Charged tRNA then participates in polypeptide synthesis and translation on ribosomes throughout its cellular lifespan (Fig. 1) [97, 98]. Mature tRNAs are notably stable molecules, exhibiting half-lives that span from several hours to days within mammalian cells. This inherent stability enables their participation in multiple cycles of translation elongation prior to degradation and holds significant implications for the persistence of tRNA modification patterns, especially under conditions of cellular stress or during the dynamic regulation of modifying enzymes [10, 99].

### tRNA modifications in the tRNA core

tRNA typically composes of 70 to 90 nucleotides, characterized by a cloverleaf secondary structure containing an acceptor stem, the D-loop (dihydrouridine loop), the anticodon loop, the variable loop, and the T-loop (thymidine, pseudouridine, and cytidine-containing or TΨC loop) in the 5' to 3' direction [100–102]. These stem loops fold into a classic three-dimensional L-shaped tertiary architecture, in which one domain consists of the acceptor stem and T-loop, while the second domain is formed by the D-loop and anticodon loop [103, 104].

Accumulating evidence indicates that modifications in the tRNA core region contribute to the formation of secondary and tertiary interactions necessary for tRNA folding, and these modifications are vital for maintaining the tRNA structure, stability, and preventing tRNA decay [105–108]. The influence of modifications on tRNA structure depends on their type and location, which includes effects on the hydrophobic properties of a base, base pairing and stacking, and stabilization of nucleotide charges [109, 110]. For example, 1-methyl-adenosine modifications at site 9 (m^1^A_9_) of mt-tRNA^Lys^ disrupt the formation of Watson-Crick base pairs within the stem, consequently hindering the development of the secondary structure [111]. Furthermore, it is worth noting that while some modifications might not impact the overall structure, they can modulate local dynamics and help align the secondary and tertiary structures of tRNA [110, 112].

### tRNA modifications at anticodon loop

The anticodon triplet in tRNA, located at positions 34, 35, and 36, is a crucial component that recognizes a specific codon on mRNA through hydrogen bonding within the ribosome [102, 113]. In codon-anticodon interaction, the first and second nucleotides of codon pair with the third and second positions of tRNA anticodon. This step is integral for translation accuracy, as it allows the tRNA to correctly identify the corresponding codon and link it to the specific amino acid attached to its 3′-end via base pairing [114, 115]. However, the base pairing between the third codon nucleotide and the first position (position 34) of anticodon does not always follows the canonical Watson-Crick base pairing, allowing unconventional pairings known as ‘wobble pairing’ [116, 117]. According to the wobble hypothesis, the 61 sense codons that encode amino acids in the standard genetic code can be deciphered by a limited number of tRNAs through various types of wobble pairs, with a diverse catalog of modified nucleosides at position 34 serving as key regulators of wobble pairing [118, 119].

To date, >30 types of chemical modifications have been documented at the wobble position [21, 120–122]. These wobble modifications exert opposing effects on decoding. For example, inosine (I_34_) expands codon recognition by permitting non-canonical (wobble) base pairing with A, C, or U at the third codon position, thereby enhancing decoding flexibility [123]. Conversely, other modifications, notably 5-methoxycarbonylmethyl-2-thiouridine (mcm^5^s^2^U_34_), restrict base pairing to prevent misreading of near-cognate codons, thereby enforcing translational fidelity [124]. This dual functionality ensures both sufficient coverage of the genetic code and accurate protein synthesis. Aside from the wobble modifications, position 37 is also a key site for tRNA modification [125, 126]. The nucleotide at position 37 is primarily a purine, and its modification creates a stacking interaction with the nucleotide at position 36 of anticodon, thereby stabilizing the structure of anticodon stem-loop (ASL) [127]. This stabilization is indispensable for compensating for variations in the ASL sequence, allowing it to dock into the ribosome decoding site with consistent speed and accuracy during polypeptide synthesis [128, 129]. A deficiency in the modification at position 37 results in ribosome stalling and +1 frameshifting [130]. In the anticodon loop, modifications influence codon-specific translation of oncogenes by impacting the decoding process [122].

### Mitochondrial tRNAs and mitochondrial tRNA modifications

**Fig. 2:**
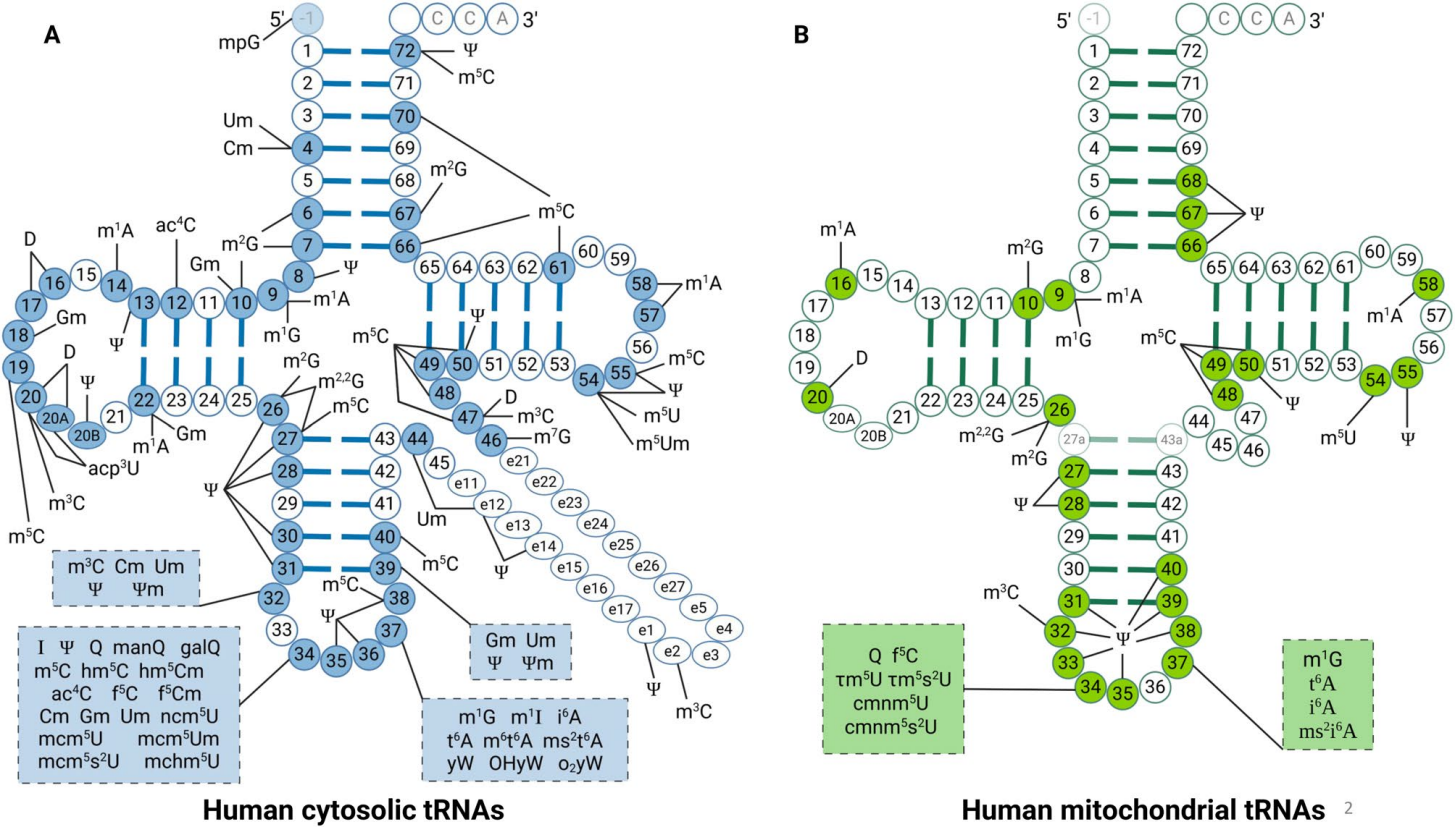
A holistic perspective on the modifications of human tRNA. (**A**) Modifications of human cytoplasmic transfer RNAs (tRNAs). (**B**) Modifications of human mitochondrial tRNAs. Nucleotides subjected to modification are highlighted in color. The nucleotide at position −1 in tRNA^His^ is added post-transcriptionally. The abbreviations for each RNA modification are in accordance with the RNA modification database MODOMICS [6]. ac^4^C, *N*^4^-acetylcytidine; acp^3^U, 3-(3-amino-3-carboxypropyl)uridine; Cm, 2′-*O*-methylcytidine; cmnm^5^s^2^U, 5-carboxymethylaminomethyl-2-thiouridine; cmnm^5^U, 5-carboxymethylaminomethyluridine; D, dihydrouridine; f^5^C, 5-formylcytidine; f^5^Cm, 5-formyl-2′-*O*-methylcytidine; galQ, galactosyl-queuosine; Gm, 2′-*O*-methylguanosine; hm^5^C, 5-hydroxymethylcytidine; hm^5^Cm, 2′-*O*-methyl-5-hydroxymethylcytidine; I, inosine; i^6^A, *N*^6^-isopentenyladenosine; m^1^A, 1-methyladenosine; manQ, mannosyl-queuosine; m^3^C, 3-methylcytidine; m^5^C, 5-methylcytidine; mchm^5^U, 5-(carboxyhydroxymethyl)uridine methyl ester; mcm^5^s^2^U, 5-methoxycarbonylmethyl-2-thiouridine; mcm^5^U, 5-methoxycarbonylmethyluridine; mcm^5^Um, 5-methoxycarbonylmethyl-2′-*O*-methyluridine; m^1^G, 1-methylguanosine; m^2^G, *N*^2^-methylguanosine; m^2,2^G, *N*^2^,*N*^2^-dimethylguanosine; m^7^G, 7-methylguanosine; m^1^I, 1-methylinosine; mpG, 5′-methylphosphoguanosine; ms^2^i^6^A, 2-methylthio-*N*^6^-isopentenyladenosine; ms^2^t^6^A, 2-methylthio-*N*^6^-threonylcarbamoyladenosine; m^6^t^6^A, *N*^6^-methyl-*N*^6^-threonylcarbamoyladenosine; m^5^U, 5-methyluridine; m^5^Um, ′-*O*-methyl-5-methyluridine; ncm^5^U, 5-carbamoylmethyluridine; OHyW, hydroxywybutosine; o_2_yW, peroxywybutosine; Q, queuosine; t^6^A, *N*^6^-threonylcarbamoyladenosine; um, 2′-*O*-methyluridine; τm^5^U, 5-taurinomethyluridine; τm^5^s^2^U, 5-taurinomethyl-2-thiouridine; Ψ, pseudouridine; Ψm, 2′-*O*-methylpseudouridine

Mitochondria are essential eukaryotic organelles for producing cellular energy and meeting metabolic needs within a cell [131–133] and they possess their own tRNAs. The human mitochondria encodes 22 mt-tRNAs and they follow a non-universal genetic code [134]. A total of 18 types of chemical modifications are detected at 137 positions of mt-tRNAs, six of these being exclusive to mt-tRNAs and absent in cyto-tRNAs (Fig. 2) [42]. These modifications are critical for translation, as they influence tRNA stability, structure, and mRNA binding, and can be dynamically affected by the metabolic environment [135]. Hypomodification of mitochondrial tRNA caused by pathogenic mutations in mitochondrial tRNA genes or in nuclear genes that encode editing enzymes can lead to severe mitochondrial diseases in humans [136–138]. Importantly, mt-tRNA-modifying enzymes are deregulated in cancer [29, 66, 139]. Studies have established a link between disrupted mt-tRNA modification and oncogenesis, inferring that these alterations contribute to cancer by hindering mitochondria function and altering the expression of key proteins involved in cell growth and survival [40, 140, 141].

### tRNA-derived RNAs (tdRs) and tRNA modifications

**Fig. 3:**
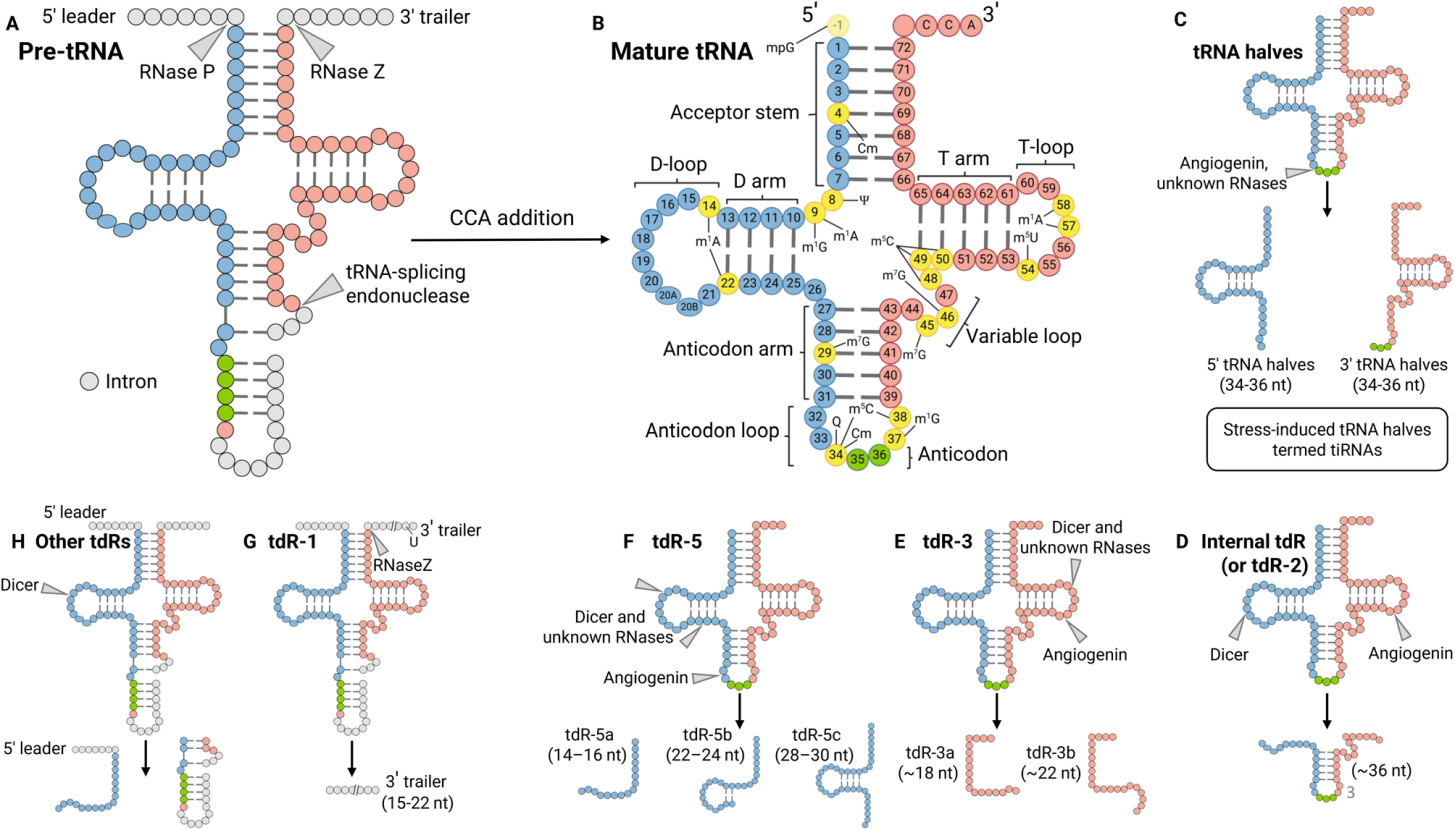
tRNA modification involved in tdR biogenesis and classification of tdR types. (**A**) Ribonucleases that cleave precursor transfer RNAs (pre-tRNAs) to generate mature tRNA. (**B**) tRNA modifications engaged in biogenesis of tRNA-derived fragments (tdRs). (**C**) The 5' and 3' halves of tRNA represent a distinct class of small RNAs produced through cleavage by angiogenin and other yet-to-be-identified ribonucleases at the anticodon loop of mature tRNA in physiological and stress conditions. (**D**) Internal tdRs are derived by the enzymatic activities of angiogenin and dicer, consisting of anticodon loop region of tRNAs while lacking the conventional 5′ and 3′ terminal sequences. (**E**) tdR-3s are generated by angiogenin, dicer and other, unknown RNases. They are derived from the 3' CCA end of mature tRNAs. (**F**) tdR-5s are generated from the 5′ end of the mature tRNA and can extend into D-loop or the anticodon stem. (**G**) tdR-1 molecules are biosynthesized by RNaseZ and originate from the 3′ trailer region of pre-tRNAs, encompassing poly(U) termination signal associated with RNA polymerase III. (**H**) Other and less prominent groups of tdRs with annotations that are uncharacterized. Cm, 2′-*O*-methylcytidine; m^1^A, 1-methyladenosine; m^5^C, 5-methylcytidine; m^1^G, 1-methylguanosine; m^7^G, 7-methylguanosine; mpG, 5′-methylphosphoguanosine; m^5^U, 5-methyluridine; nt, nucleotide; Q, queuosine; Ψ, pseudouridine

In 1979, Speer and colleagues first identified seven types of tRNA decomposition products in the urine of cancer patients from 13 different malignancies [142]. In human cells, tRNA-derived RNAs (tdRs) are produced from mature or precursor tRNAs through cleavage by specific endonucleases. These include angiogenin (ANG or RNase 5), which primarily generates stress-induced fragments (tiRNAs) by cleaving in the anticodon loop. The enzyme Dicer is responsible for generating smaller fragments tdR-3s and tdR-5s. RNase P cleaves at position 7, 8 to remove the leader sequence, while RNase Z (ELAC2) processes the 3' trailer of precursor tRNAs to produce tdR-1s. Additionally, RNase 1 has been implicated in the production of particular tdRs under specific conditions [143–145]. tdRs are categorized into six distinct classes according to their length and the source of their parental tRNAs, with more than 20,000 unique tdRs that have been identified to date (Fig. 3) [47, 146–151].

Accumulating evidence implicates that tdRs are not just mere debris of tRNA degradation, but possess regulatory roles [152–154]. Translation control is the most extensively studied function of tdRs, which predominantly inhibit protein biosynthesis in response to various stimuli [55, 153, 155–161]. For example, Ivanov et al. showed that selected tiRNAs suppress protein biosynthesis by displacing eukaryotic translation initiation factor 4 G (eIF4G) and eIF4A from uncapped RNAs more effectively than from capped RNAs, whilst also displacing eIF4F from the isolated 7-methylguanosine (m^7^G) cap, thereby disrupting a crucial and rate-limiting step in canonical cap-dependent translation initiation [155]. However, tdRs can paradoxically promote translation under certain conditions, as exemplified by a specific tdR-3 derived from tRNA^Leu^_CAG_ which directly interacts with the mRNAs of two ribosomal protein (RPS28 and RPS15), thereby enhancing their translation and facilitating ribosome biogenesis [58, 162, 163]. Apart from their role in translation, tdRs are also engaged in post-transcriptional regulation of gene expression [57, 164–170]. First, tdRs exert their influence through mechanisms resembling that of microRNAs. tdRs form complexes with Argonaute (AGO) proteins that interact with 3' untranslated regions (3'UTR) of target mRNAs, thereby suppressing gene expression [164]. Besides, tdRs silence gene expression by interacting with P-element induced wimpy testis (PIWI) proteins, a subgroup of AGO family [165–167]. tdRs also indirectly modulate gene expression via interaction with RNA-binding proteins (RBPs) [171–175]. Goodarzi et al. revealed a novel category of tdRs derived from tRNA^Tyr^, tRNA^Gly^, tRNA^Glu^, and tRNA^Asp^ that can modulate oncogene expression through the RBP Y-box binding protein 1 (YBX1), ultimately inhibiting cancer metastasis [171].

The crosstalk between tRNA modifications and tdRs biogenesis is bidirectional and functionally critical. On one hand, modifications can protect tRNAs from cleavage. For example, methylation of tRNAs, such as 5-methylcytidine (m^5^C) installed by NOP2/Sun RNA methyltransferase 2 (NSUN2), stabilizes their secondary and tertiary structures. This structural rigidity protects mature tRNAs from cleavage by ANG under stress conditions [176]. Conversely, the absence of particular modifications results in hypo-modified tRNAs, serving as a signal for cleavage. The lack of m^5^C, for example, enhances tRNA vulnerability to ANG-mediated cleavage, which subsequently causes the accumulation of tdRs capable of reprogramming translation [177]. Similarly, the demethylation of m^1^A by AlkB homolog 3 (ALKBH3) has been shown to increase tRNA sensitivity to ANG cleavage, generating tdRs that promote cancer cell survival [178]. On the other hand, the modification pattern on tdRs themselves dictates their stability and function. For instance, m^1^A methylation within the seed region of a tdR-3b in bladder cancer disrupts its base-pairing capacity with target mRNAs, thereby modulating its gene-silencing activity and promoting oncogenic phenotypes [168]. Pseudouridylation (Ψ) of a specific class of tdRs (mTOGs) by pseudouridine synthase 7 (PUS7) is essential for their ability to repress protein synthesis, and its loss contributes to leukemogenesis [179]. Thus, the modification status is a key determinant of both tdR biogenesis and their subsequent biological activity, establishing a complex regulatory network that bridges tRNA processing and gene expression control.

## The realm of tRNA modifications

**Fig. 4:**
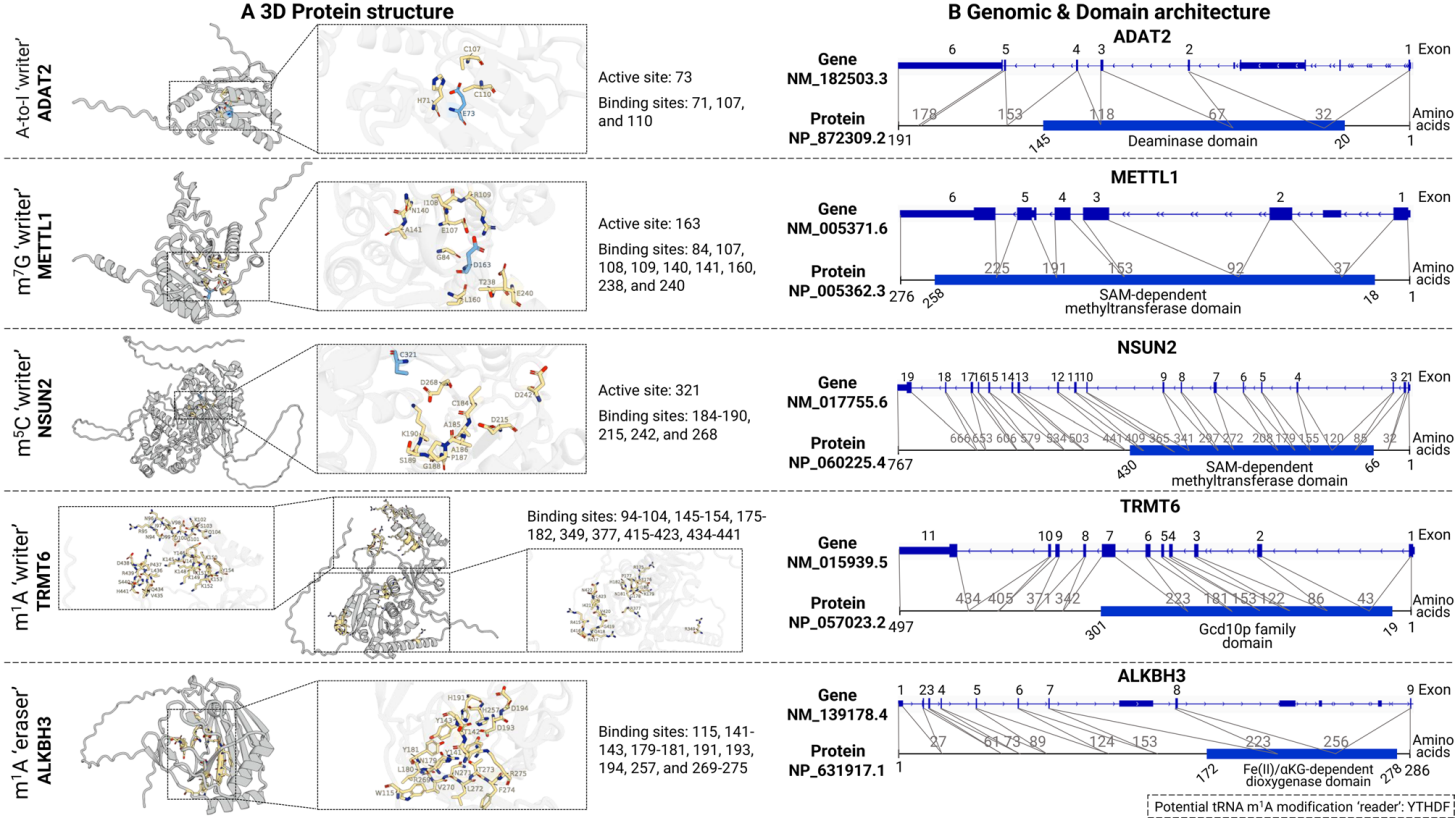
Genomic and protein architectures of representative tRNA modification enzymes in cancer. (**A**) Three-dimensional structures of tRNA-modifying enzymes. For ADAT2, METTL1, NSUN2, TRMT6, and ALKBH3, the overall structure (left) and a close-up view (right) are shown. Critical residues and surrounding regions are visualized with highlighted distinct colors. (**B**) Integrated genomic and protein domain architecture. For each enzyme, the exon-intron organization is displayed above the corresponding protein domain architecture. Connecting lines indicate which exons encode specific protein regions, illustrating how genomic structure maps to protein function. Structural models were generated using AlphaFold3 (accessed via UniProt database), and genomic coordinates were derived from NCBI’s MANE select transcripts. ADAT2, adenosine deaminase tRNA specific 2; ALKBH3, AlkB homolog 3; METTL1, methyltransferase-like 1; NSUN2, NOL1/NOP2/Sun 2; TRMT6, tRNA methyltransferase 6

Transfer RNA (tRNA) modifications constitute a vital aspect of epitranscriptomic regulation that significantly impacts the stability, folding, decoding proficiency, and translational precision of tRNA molecules. These chemical modifications, which are introduced post-transcriptionally at multiple sites within tRNA, are mediated by specific enzymatic sets and are crucial for facilitating accurate and efficient protein synthesis. The subsequent sections offer a comprehensive overview of the principal types of tRNA modifications, their biosynthetic pathways, and their functional importance across diverse biological contexts (Table 1; Fig. 4).

**Table 1:** The landscape of tRNA modifications: chemical structure, positions, enzymes, and functional roles

Modification type	Chemical structure	Structure description	Common position(s)	Catalytic enzymes	Key functions	Notes/Specific tRNAs
*Adenosine variants*						
Inosine (I)		Deamination of adenosine, replacing an amine group (−NH2) with a keto group (=O) at position 6 of the adenine base, resulting in inosine	Anticodon wobble position 34	ADAT2/ADAT3 (hetADAT)	Enhances wobble pairing (decodes A, C, U-ending codons)	In eight distinct tRNAs: tRNA^Leu^_AAG_, tRNA^Arg^_ACG_, tRNA^Ser^_AGA_, tRNA^Pro^_AGG_, tRNA^Ala^_AGC_, tRNA^Val^_AAC_, tRNA^Ile^_AAT_ and tRNA^Thr^_AGT_
1-methylinosine (m^1^I)		Inosine with a methyl group attached to the nitrogen at position 1 (N1) of the hypoxanthine base	Position 37 (adjacent to anticodon)	ADAT1, Trm5	Suppresses frameshifts, improves translational precision	Eukaryotic tRNA^Ala^
1-methyladenosine (m^1^A)		Methylation at the nitrogen at position 1 (N1-CH₃) of the adenine base	D-loop, D arm, and T-loop: 9, 14, 22, 57, 58 (cyto); 9, 58 (mt)	TRMT6, TRMT61A, TRMT61B, TRMT10	Modulates tRNA processing, stability, protein interactions, mitochondrial activity	Regulated by writers, readers (YTHDF), erasers (ALKBH1/3, FTO)
*N*^6^-methyladenosine (m^6^A)		Methylation at the N6 position of the adenine base	Position 37 (adjacent to anticodon)	TrmM (also known as YfiC)	Proposed to stabilize codon-anticodon interactions	Relatively rare in tRNA Mostly observed in bacteria
*N*^6^-isopentenyladenosine (i^6^A)		Adenosine with an isopentenyl (prenyl) group (-CH₂-CH = C(CH₃)₂) attached to the N6 atom of the adenine base	Position 37 (adjacent to anticodon)	MiaA (bact.), TRIT1 (humans)	Fine-tunes RNA-cleaving active sites	Precursor to ms^2^i^6^A/ms^2^io^6^A
2-methylthio-*N*^6^-isopentenyladenosine (ms^2^i^6^A)		Adenosine with a methylthio group (-SCH₃) at the C2 position and an isopentenyl group (-CH₂-CH = C(CH₃)₂) at the N6 position of the adenine base	Position 37 (adjacent to anticodon)	MiaB, CDK5RAP1 (mt)	Promotes efficiency and accuracy of mitochondrial translation	Human mt-tRNAs for Ser, Phe, Tyr, Trp
*N*^6^-threonylcarbamoyladenosine (t^6^A)		Adenosine with an N6-threonylcarbamoyl moiety (L-threonine linked via a carbamoyl bridge, -NH–C(O)–NH–) attached to the N6 atom of the adenine base	Position 37 (adjacent to anticodon)	YRDC, KEOPS complex (GON7, LAGE3, OSGEP, TP53RK, TPRKB)	Stabilizes ASL, facilitates codon pairing, maintains fidelity	Universal in ANN-decoding tRNAs
2-methylthio-*N*^6^-threonylcarbamoyladenosine (ms^2^t^6^A)		Adenosine bearing a methylthio group (-SCH₃) at the C2 atom and an N6-threonylcarbamoyl moiety (L-threonine linked via a carbamoyl bridge, -NH–C(O)–NH–) attached to the N6 atom of the adenine base	Position 37 (adjacent to anticodon)	CDKAL1	Prevents misreading of cognate codons	Found in tRNA^Lys^
*Cytidine modifications*						
3-methylcytidine (m^3^C)		Methylation at the nitrogen at position 3 (N3-CH₃) of the cytosine base	32 (anticodon loop), 20 (D-loop), 47:3 (variable loop)	METTL2A/B, METTL6, METTL8	Optimizes tRNA stability, maintains fidelity and efficiency	Eraser: ALKBH3
5-methylcytidine (m^5^C)		Methylation at the carbon at position 5 (C5-CH₃) of the cytosine base	Anticodon loop, variable loop, T arm, and acceptor stem: 34, 38, 48, 49, 50, 72	NSUN2, NSUN3, NSUN4, NSUN6, DNMT2	Augments translation, modulates tRNA stability, stress response	Erasers: TET family, ALKBH1
*N*^4^-acetylcytidine (ac^4^C)		Cytidine with an acetyl group attached via an amide linkage to the N4 atom of the cytosine base	Wobble of Met; D-arm of Ser, Leu	NAT10 (with THUMPD1)	Enhances fidelity, thermal resilience, modulates tRNA biogenesis/degradation	tRNA^Ser^
5-formylcytidine (f^5^C)		Oxidation of the methyl group of m^5^ C to a formyl group (C5-CHO)	Anticodon wobble position 34 (mt)	NSUN3, ALKBH1	Augments recognition of purine-ending codons	mt-tRNA^Met^
*Uridine modifications*						
Dihydrouridine (D)		Uridine with a saturated 5,6-double bond in the uracil base, resulting in a 5,6-dihydrouracil ring	D-loop and variable loop: 16/17, 20, 20a/b, 47	Dihydrouridine synthases (DUS)	Increases local flexibility, stabilizes tertiary interactions	Found in D-loop and variable loop
5-methyluridine (m^5^U)		Uridine with a methyl group attached to the carbon at position 5 (C5) of the uracil base	Position 54 (T-loop)	TRMT2A (cyto), TRMT2B (cyto/mt)	Evolutionarily conserved function	Widely distributed
5-taurinomethyluridine (τm^5^U)		Addition of a taurine-containing side chain at the C5 position of uracil	Anticodon wobble position 34 (mt)	GTPBP3, MTO1	Enables precise anticodon-codon interactions, ensures fidelity	mt-tRNA^Leu^(UUR), mt-tRNA^Trp^
5-taurinomethyl-2-thiouridine (τm^5^s^2^U)		Uridine modified with a taurinomethyl group (-CH₂-NH-CH₂-CH₂-SO₃H) attached to the C5 atom and a thio group (=S, replacing the carbonyl oxygen) at the C2 atom of the uracil base	Anticodon wobble position 34 (mt)	TRMU (MTU1), NFS1	Enables precise anticodon-codon interactions, ensures fidelity	mt-tRNA^Lys^, mt-tRNA^Glu^, mt-tRNA^Gln^
5-carboxymethylaminomethyluridine (cmnm^5^U)		Uridine with a carboxymethylaminomethyl group (-CH₂-NH-CH₂-COOH) attached to the C5 atom of the uracil base	Anticodon wobble position 34 (mt)	Dynamically regulated	Proposed functional analog to τm^5^U under low taurine	mt-tRNA^Lys^, mt-tRNA^Glu^, mt-tRNA^Gln^
*Guanosine & Pseudouridine*						
1-methylguanosine (m^1^G)		Guanosine with a methyl group attached to the nitrogen at position 1 (N1) of the guanine base	9 (D arm), 37 (anticodon loop)	TRMT10A (G_9_), TRMT5 (G_37_)	m^1^G_9_: maintains structure; m^1^G_37_: prevents frameshifting	Widely distributed
*N*^2^,*N*^2^-dimethylguanosine (m^2,2^G)		Addition of two methyl groups to the exocyclic nitrogen (N2-(CH₃)₂) attached to carbon 2	26 (D arm), 27 (anticodon arm)	TRMT1, TRMT1L	Maintains stability	Eraser: ALKBH7 (regulates mt-tRNA processing)
2′-*O*-methylguanosine (Gm)		Guanosine with a methyl group attached to the 2′-hydroxyl (2′-O) of the ribose moiety	18 (D loop), 34 (wobble position)	TARBP1 (G_18_), FTSJ1/WDR6 (G_34_)	Gm_34_: facilitates UUU codon translation efficiency	FTSJ1 modifies cyto-tRNA^Phe^_GAA_
7-methylguanosine (m^7^G)		Methylation at the nitrogen at position 7 (N7-CH₃) of the guanine base, introducing a positive charge	Position 46 (variable loop)	METTL1/WDR4 complex	Structural stabilization, ribosome interaction, codon usage regulation	Widely distributed
Queuosine (Q)		Hypermodified nucleoside consisting of 7-deazaguanosine linked to a cyclopentenediol moiety via an aminomethyl group at the 7-position	Anticodon wobble position 34	QTRT1/QTRT2 (TGT)	Enhances C-ending, reduces U-ending codon translation; prevents ER stress	Diet/gut microbiome derived; tRNAs for Asn, Asp, His, Tyr
Pseudouridine (Ψ)		Isomerization of uridine where the base is attached to the ribose via a carbon-carbon bond (C1’−C5’) instead of a nitrogen-carbon bond	55 (TΨC loop), 13 (D arm), 27/28 (anticodon arm), 38/39 (anticodon loop/arm)	Pseudouridine synthases (PUSs, e.g., TRUB1 in mt)	Stabilizes tRNA structure	Most prevalent RNA modification

^*^ Abbreviation ASL, anticodon stem-loop; cyto, cytoplasmic; ER, endoplasmic reticulum; mt, mitochondrial

### Adenosine variants: writers, erasers, and functional impacts

#### Inosine (I)/1-methylinosine (m^1^I)

In humans, inosine (I) at position 34 of tRNA is generated post-transcriptionally through deamination of adenosine (A) that occurs in 8 distinct tRNAs: tRNA^Leu^_AAG_, tRNA^Arg^_ACG_, tRNA^Ser^_AGA_, tRNA^Pro^_AGG_, tRNA^Ala^_AGC_, tRNA^Val^_AAC_, tRNA^Ile^_AAT_ and tRNA^Thr^_AGT_ [180, 181]. This modification is orchestrated by the heterodimeric adenosine deaminase (known as hetADAT), comprising the catalytic subunit adenosine deaminase tRNA specific 2 (ADAT2) and the tRNA-binding subunit ADAT3 [182]. The I_34_-tRNA modification enhances the wobble-pairing flexibility of the anticodon, allowing I_34_-tRNAs to recognize synonymous codons with A, C, or U endings as compared to A_34_-tRNAs that only effectively pair with U-ended codons [117, 123]. Inosine at positions 37 and 57 of tRNA are additional modified to form methylated variants (m^1^I_37_, m^1^I_57_) [183, 184]. 1-methylinosine 37 (m^1^I_37_) is exclusive to eukaryotic tRNA^Ala^ and is generated by ADAT1 and tRNA methyltransferase 5 (Trm5) [185, 186]. m^1^I_37_ is believed to suppress translational frameshifts while improving translational precision [187]. Adenosine at position 57 (A_57_) undergoes conversion to m^1^I_57_ through a two-step reaction, and it can also be modified into di-methylated inosine (1,2’-*O*-dimethylinosine, m^1^Im_57_) in hyperthermophilic species [188, 189]. m^1^I_57_ or m^1^Im_57_ is found only in archaeal tRNA^Ile^ and their function remains unknown [190].

#### 1-methyladenosine (m^1^A)

m^1^A modification, involving the transfer of methyl group to N1 position of adenine, is the most prevalent methylated nucleoside in tRNAs [191]. In cyto-tRNA, m^1^A modification is found at five distinct positions (9, 14, 22, 57 and 58), and m^1^A modification at two of these positions (9 and 58) are present in mt-tRNAs [192]. m^1^A modification is tightly regulated by ‘writers’, ‘readers’, and ‘erasers’, which add, recognize and remove m^1^A on tRNAs, respectively [193, 194]. m^1^A methylation ‘writers’ consist of tRNA methyltransferase 6 (TRMT6), TRMT61A, TRMT61B, and TRMT10 [195–198]. ‘readers’ that recognize m^1^A methylation include YTH domain-containing proteins, including YTHDF1, YTHDF2, YTHDF3, and YTHDC1 [199]. AlkB homolog 1 (ALKBH1), ALKBH3, ALKBH7, and fat mass and obesity-associated protein (FTO) function as the ‘erasers’ responsible for m^1^A demethylation [200–203]. This modification in tRNA is critical for the modulation of tRNA processing, stability, protein interactions, and mitochondrial activity [192, 204, 205].

#### *N*^6^-isopentenyladenosine (i^6^A)/2-methylthio-*N*^6^-isopentenyladenosine (ms^2^i^6^A), 2-methylthio-*N*^6^-(cis-hydroxy)isopentenyl adenosine (ms^2^io^6^A)

i^6^A possesses an isopentenyl (prenyl) group at the N6 position of adenine and functions as the precursor for all isopentenyl-based hypermodifications, including ms^2^i^6^A and ms^2^io^6^A [206, 207]. Isopentenyl-tRNA transferases have been identified in bacteria (MiaA), yeast (Mod5 and Tit1), nematodes (GRO-1) and mammals (TRIT1) [208–211]. i^6^A-modified nucleotide at position 37 adjacent to anticodon fine-tunes the active site of RNA-cleaving deoxyribozymes, leading to a shift of the cleavage site [212, 213]. The hypermodified nucleoside ms^2^i^6^A is synthesized through the action of MiaB, which catalyzes the final step involving the attachment of a methylthio (–SCH_3_) group at the C2 position of i^6^A_37_ in tRNAs containing adenine at position 36 of the anticodon [214–217]. Human orthologue Cdk5 regulatory subunit-associated protein 1 (CDK5RAP1) is localized in the mitochondria and is involved in the modification of Ser (AGN), Phe, Tyr, and Trp codons in mt-tRNAs at i^6^A_37_ [218]. This modification promotes efficiency and accuracy of translation in mitochondria [219].

#### *N*^6^-threonylcarbamoyladenosine (t^6^A)/*N*^6^-methyl-*N*^6^-threonylcarbamoyladenosine (m^6^t^6^A)/2-methylthio-*N*^6^-threonylcarbamoyladenosine (ms^2^t^6^A)

t^6^A modification is universally present at position 37 of ANN-decoding tRNAs (where N represents A, U, C, and G) across the three domains of life, with the incorporation of *L*-threonine through a ureido linkage at N6 nitrogen of adenosine [220–222]. In humans, t6A biosynthesis pathway consists of two steps that involve multiple enzymes, including yrdC N^6^-threonylcarbamoyltransferase domain containing (YRDC) enzyme and kinase, endopeptidase and other proteins of small size (KEOPS) protein complex, which is composed of five subunits—GON7 subunit of KEOPS complex (GON7), L antigen family member 3 (LAGE3), O-sialoglycoprotein endopeptidase (OSGEP), tumor protein p53 regulating kinase (TP53RK), and TP53RK binding protein (TPRKB)—arranged in a linear sequence [223–225]. t^6^A stabilizes structural configurations of the ASL, facilitates anticodon-codon pairing, and maintains translational fidelity [226, 227]. *N*^6^-methylation of t^6^A (m^6^t^6^A) is a post-transcriptional modification located at position 37 of tRNAs derived from bacteria, insects, plants, and mammals [184]. This modification is catalyzed by TRMO utilizing S-adenosyl-*L*-methionine as a methyl donor, resulting in the formation of m^6^t^6^A in tRNA^Thr^ specifically for ACY codons [228]. m^6^t^6^A has been shown to potentiate the attenuation activity of the *thr* operon, indicating that TRMO plays a crucial role in ensuring the efficient decoding of ACY [228]. Methylthiolation of t^6^A to form ms^2^t^6^A in tRNA is catalyzed by CDKAL1 threonylcarbamoyladenosine tRNA methylthiotransferase (CDKAL1) at position 37 of tRNA^Lys^. It functions to prevent the misreading of its cognate codons particularly when the translation rate is relatively high [229–232].

### Cytidine modifications: epitranscriptomic control of translation

#### 3-methylcytidine (m^3^C)/5-methylcytidine (m^5^C)

m^3^C is predominantly located at position 32 within anticodon loop, and it is also present at position 47:3 in variable loop and position 20 in D-loop [233, 234]. The methyltransferase-like (METTL) family, including METTL2A/B, METTL6, and METTL8, serves as ‘writers’ by adding a methyl group to N3 of cytosine at specific positions in tRNA [235, 236], while ALKBH3 functions as a demethylase [200, 233]. m^3^C modification plays a significant role in optimizing tRNA structural stability and safeguarding translation fidelity and efficiency [237–240]. In mammals, m^5^C modifications have been detected at positions 34, 38, 48, 49, 50, and 72 of tRNA [241, 242]. m^5^C modifications are orchestrated by members of NOL1/NOP2/Sun (NSUN) protein family, comprising NSUN2, NSUN3, NSUN4, and NSUN6, alongside the DNA methyltransferase 2 (DNMT2) [243–246]. m^5^C modification not only augments the efficiency and precision of translation but also modulates tRNA stability, thereby exerting a profound influence on cellular metabolism and the organism’s response to stress [60, 235, 247–249]. The primary ‘erasers’ consist predominantly of the ten-eleven translocation (TET) family and ALKBH1 [250–252].

#### *N*^4^-acetylcytidine (ac^4^C)

ac^4^C modification is observed at the wobble position of tRNA^Met^ and within the D-arm of both tRNA^Ser^ and tRNA^Leu^ [253, 254]. The formation of ac^4^C modifications is catalyzed by *N*-acetyltransferase 10 (NAT10), with the assistance of thiouridylase methyltransferase and pseudouridine synthase domain-containing protein 1 (THUMPD1) [253, 255, 256]. ac^4^C-modified sites in tRNA not only enhances the fidelity of protein translation and contributes to thermal resilience of organisms, but also modulates the biosynthesis and degradation of specific tRNA molecules [257–259].

#### Cytidine derivatives: 2′-*O*-methylcytidine (Cm)/5-formylcytidine (f^5^C) and 5-formyl-2′-*O*-methylcytidine (f^5^Cm)/5-hydroxymethylcytidine (hm^5^C) and 2′-*O*-methyl-5-hydroxymethylcytidine (hm^5^Cm)

2′-*O*-methylation (Nm) is a ubiquitous tRNA modification through the addition of a methyl group to 2′ hydroxyl of the ribose moiety in nucleosides, including Cm, Um, Gm, and Ψm [260, 261]. Trm13 facilitates the modification of Cm at the fourth position of tRNA^Gly^ [262]. 2′-*O*-methylation of the wobble position in human elongator tRNA^Met^_CAT_ is achieved by concerted action of a nucleolar and a CB-specific box C/D ribonucleoprotein complex, which incorporates the small nucleolar RNA, C/D box 97 (SNORD97) and SNORD133 (also known as SCARNA97) guide RNAs to mediate this modification [263]. f^5^C modification, located at position 34 of mt-tRNA^Met^, is synthesized through a multistep process mediated by NSUN3 and then ALKBH1, thereby augmenting its ability to recognize purine-ending codons [244, 264–266]. The presence of f^5^Cm has been identified at the wobble position of mammalian cyto-tRNA^Leu^ bearing CAA anticodon, and it is postulated that f^5^Cm facilitates recognition of UUA in addition to UUG [252, 267]. hm^5^C, formed by oxidation of m^5^C by ALKBH1, undergoes further conversion to f^5^C [252]. Reduction of f^5^C_34_ in tRNA^Met^ to hm^5^C enables the resultant hydroxyl group to maintain an intramolecular hydrogen bond with amino group, thereby preserving tertiary structure [268]. hm^5^Cm, a reduced variant of f^5^Cm, has also been identified in tRNA^Leu^ [252, 267].

### Uridine diversity: wobble position architects

#### Dihydrouridine (D)

Dihydrouridine (D, or DHU) RNA modification, a modified pyrimidine nucleoside with the corresponding nucleobase 5,6-dihydrouracil, is synthesized from uridine (U) through hydrogenation [269, 270]. In eukaryotes, D modifications are catalyzed by dihydrouridine synthase enzymes (DUS) and are primarily located in the tRNA D-loop at positions 16/17, 20, and 20a/20b, as well as at position 47 in the variable loop [6]. It is proposed that the locally increased flexibility mediated by D modifications may improve the formation of stabilizing neighboring tertiary base interactions in the elbow region of tRNA [85].

#### Methyluridines: 5-methyluridine (m^5^U)/2′-*O*-methyluridine (Um), 2′-*O*-methyl-5-methyluridine (m^5^Um)

Methylation at C5 of uridine (m^5^U) is a prevalent and evolutionarily conserved alteration found at position 54 of cyto-tRNAs in diverse species [271, 272]. In mammals, TRMT2A mediates m^5^U_54_ methylation in cyto-tRNAs, while TRMT2B is responsible for methylating m^5^U_54_ in both cytoplasmic and mitochondrial tRNAs [272, 273]. Notably, the TRMT2A-mediated m^5^U_54_ modification in tRNAs protects against cleavage by angiogenin, thereby emphasizing the significance of tRNA modifications in the production of tdRs [274]. Um is commonly found in cyto-tRNAs featuring a long variable loop such as tRNA^Ser^, and its formation is catalyzed by Trm44, an enzyme present in both metazoans and fungi [275]. m^5^Um is a derivative of Um characterized by methylation at 2′ and 5′ position of uridine, and it has been found in tRNA^Lys^ and tRNA^Glu^ from *Drosophila* and mammals [276–278].

#### Side chain-modified uridines: 3-(3-amino-3-carboxypropyl)uridine (acp^3^U)/5-taurinomethyluridine (τm^5^U), 5-taurinomethyl-2-thiouridine (τm^5^s^2^U)/5-carboxymethylaminomethyluridine (cmnm^5^U), 5-carboxymethylaminomethyl-2-thiouridine (cmnm^5^s^2^U)

Biosynthesis of acp^3^ U entails the transfer of 3-amino-3-carboxypropyl (acp) group from S-adenosylmethionine (SAM) to N3 of uracil base [279, 280]. In human cells, biosynthesis of acp^3^ U_20_ by DTW Domain Containing 1 (DTWD1) and acp^3^ U_20a_ by DTWD2 is required, as the loss of either enzyme can lead to dihydrouridine formation at positions D_20_ or D_20a_, thereby implying that acp^3^ U modification inhibits D_20(a)_ synthesis [281, 282]. While taurine is predominantly classified as a free amino acid, recent work has identified its presence in mitochondrial tRNAs [283–285]. τm^5^U is found in mt-tRNA^Leu^_UUR_ and mt-tRNA^Trp^, whereas τm^5^s^2^U is present in mt-tRNA^Lys^, mt-tRNA^Glu^, and mt-tRNA^Gln^ [42]. GTP binding protein 3 (GTPBP3) together with mitochondrial translation optimization 1 protein (MTO1) catalyzes τm^5^U synthesis. Whereas τm^5^s^2^U is catalyzed by mitochondrial tRNA-specific 2-thiouridylase 1 (TRMU or MTU1) in conjunction with NFS1 cysteine desulfurase (NFS1) [285]. These modifications are crucial for enabling precise anticodon-codon interactions, thus ensuring the fidelity of mitochondrial protein synthesis [286, 287]. cmnm^5^U and its derivative cmnm^5^s^2^U, characterized by the replacement of taurine moiety in m^5^U with glycine, have recently been identified in human mitochondrial tRNAs [42]. Given the structural similarities between taurine and glycine, it is plausible that glycine is incorporated in place of taurine under taurine-depleted conditions [21]. In alignment with this, the presence of the cmnm^5^U modification in tRNAs derived from taurine-starved cells implies that tRNA modification is dynamically regulated by taurine availability [284].

#### Wobble U_34_ hypermodifications: 5-methoxycarbonylmethyluridine (mcm^5^U), 5-methoxycarbonylmethyl-2′-*O*-methyluridine (mcm^5^Um), 5-carbamoylmethyluridine (ncm^5^U), 5-methoxycarbonylmethyl-2-thiouridine (mcm^5^s^2^U), 5-(carboxyhydroxymethyl) uridine methyl ester (mchm^5^U)

Uridine at the wobble position in eukaryotic tRNAs typically incorporate modifications including mcm^5^U, mcm^5^Um, ncm^5^U, mcm^5^s^2^U, or mchm^5^U, which play a pivotal role in modulating decoding properties [18, 288]. mcm^5^U and mcm^5^s^2^U are associated with ‘split’ codon boxes, where codons ending in pyrimidines and purines correspond to distinct amino acids, while ncm^5^U is observed in ‘family’ codon boxes, where all 4 ending codons encode a single amino acid [289, 290]. In mammals, (*S*)-diastereomer of mchm^5^U is detected in tRNA^Gly^_UCC_, while (*R*)-mchm5U is found in tRNA^Arg^_UCG_ [291, 292]. These modifications are complex processes involving multiple proteins in elongator complex, including acetyl-transferase elongator complex proteins 1–6 (ELP1-6), ALKBH8, TRMT112, and cytosolic thiouridylase subunits 1/2 (CTU1/CTU2) [293–297]. One example of this complexity is the formation of the mcm5s^2^ U modification, which involves two genetically separable pathways [298]. The first is the Elongator pathway, which introduces the mcm^5^ (or ncm^5^) side chain at the C5 position. The second is the ubiquitin-related modifier 1 (Urm1) pathway, which introduces a thiol group at the C2 position. This latter pathway employs the Urm1 to mediate sulfur transfer from a cysteine residue to the target uridine, highlighting an ancient and sophisticated mechanism for tRNA modification [299, 300].

### Guanosine and pseudouridine: structural and regulatory modulators

#### Guanosine variants: 1-methylguanosine (m^1^G)/*N*^2^-methylguanosine (m^2^G), *N*^2^,*N*^2^-dimethylguanosine (m^2,2^G)/2′-*O*-methylguanosine (Gm)/7-methylguanosine (m^7^G)/5′-methylphosphoguanosine (mpG)

m^1^G modifications are usually present position 9 and 37, and catalyzed by TRMT10A and TRMT5, respectively [301]. As the methyl group of m^1^G disrupts G-C base pair, it is likely that m^1^G_9_ is involved in maintaining tRNA structure [22]. Data from Eubacteria suggests that m^1^G_37_ appears to prevent frameshifting [302]. AlkB is involved in the demethylation of m^1^G, however, its specific function remains largely unclear [303]. Guanosine can undergo either mono- or di-methylation at the exocyclic nitrogen attached to carbon 2, resulting in the formation of m^2^G or m^2,2^G [304]. m^2^G is found at positions 6, 7, 10, 26, and 67 in cyto-tRNAs from various species [305–308]. In human, the enzymes responsible for m^2^G_6/7_ and m^2^G_10_ modifications in cyto-tRNAs are TRMT112/THUMPD3 and TRMT11/TRMT112, respectively [309, 310]. In contrast to evolutionarily conserved and architecturally related m^2^G methyltransferases that target the acceptor stem and D-arm, enzymes mediating m^2^G_26_, m^2,2^G_26_, and m^2,2^G_27_ modifications in tRNAs are Trm1/TRMT1 and TRMT1L [311–313]. ALKBH7 is an RNA demethylase that catalyzes demethylation of m^2,2^G in nascent mt-tRNA, thereby regulating the processing of nascent polycistronic mt-tRNA and mitochondrial function [203]. A conserved guanosine at position 18 (G_18_) within D-loop of tRNAs is frequently modified to 2’-*O*-methylguanosine (Gm) through methylation of its 2′-hydroxyl group, a process that is catalyzed in prokaryotes by tRNA (Gm_18_) methyltransferase (trmH), and in lower and higher eukaryotes by trm3 and TARBP1, respectively [314, 315]. FtsJ RNA Methyl-transferase Homolog 1 (FTSJ1), in collaboration with WD repeat domain 6 (WDR6), catalyzes formation of Gm_34_ on human cyto-tRNA^Phe^_GAA_. Translation efficiency of UUU codon decoded by tRNA^Phe^_GAA_ is primarily facilitated by FTSJ1 [316–318]. m^7^G refers to methylation of N7 on guanylate, which can introduce positively charged or amphiphilic ions into nucleobases and can occur at position 46 (m^7^G_46_) within the variable loop of tRNA [67, 301]. This modification is catalyzed by METTL1/WDR4 complex, and is crucial for the proper functioning of tRNAs, including structural stabilization, interaction with ribosome, and regulation of codon usage [68, 319–321]. tRNA His guanylyltransferase (Thg1) family consists of a distinct set of 3′-5′ nucleotide addition enzymes found universally in eukaryotes, where they perform a critical role in adding a single essential G residue (G − 1) to 5′ end of tRNA^His^ for its maturation [322, 323]. Bicoid interacting 3 domain containing RNA methyltransferase (BCDIN3D) then functions as a cyto-tRNA^His^-specific 5′-methylphosphate capping enzyme, and methylation of the 5′-monophosphate on cyto-tRNA^His^ might contribute to its stability under specific conditions [324, 325].

#### Queuosine (Q)/galactosyl-queuosine (galQ) and mannosyl-queuosine (manQ)

Queuosine (Q_34_) is a hypermodified nucleoside derived from guanine, found at the wobble position of tRNAs possessing 5’-GUN-3' anticodon sequence, and is involved in decoding of Asn, Asp, His and Tyr codons (AAC/U, GAC/U, CAC/U, UAC/U, NAC/U) [326, 327]. In bacteria, queuine (q) is biosynthesized [328]; however, in eukaryotes Q is obtained from diet or gut microbiome. Eukaryotic tRNA guanine transglycosylase (TGT), consisting of a heterodimeric complex of queuine tRNA-ribosyltransferase catalytic subunit 1 (QTRT1) and QTRT2, facilitates the replacement of guanine with queuine [329–331]. Q_34_ enhances the translation of C-ending codons, but reduces translation of U-ending codons [332, 333]. Loss of Q_34_ in tRNAs impairs optimal translation, causes protein unfolding, leading to induction of endoplasmic reticulum stress and unfolded protein response, highlighting Q_34_ as a nutritionally controlled modification that optimizes translation elongation in both the cytoplasm and mitochondria in response to diet [332, 334]. In vertebrate tRNA^Tyr^ and tRNA^Asp^, queuosine is glycosylated with galactose and mannose to produce galQ and manQ, respectively, through the action of the enzymes QTGAL (queuosine-tRNA galactosyltransferase) and QTMAN (queuosine-tRNA mannosyltransferase) [335, 336], and Q-glycosylation functions to maintain proteostasis [336].

#### Pseudouridine (Ψ)/*N*^1^-methylpseudouridine (m^1^Ψ), 2′-*O*-methylpseudouridine (ψm)

Pseudouridylation, referred to as ‘fifth nucleotide’ of RNA, is widely regarded as the most prevalent post-transcriptional modification in RNA [337–339]. Ψ is a C5 glycoside isomer of uridine featuring a C1′-C5′ bond between the ribose sugar and uracil, as opposed to the typical C1′-N1′ bond [340]. Ψ is present in nearly all tRNAs within TΨC stem-loop at Ψ_55_, whereas other Ψ sites are observed less frequently at other positions, including Ψ_13_, Ψ_27/28_, and Ψ_38/39_, and they contribute to the stabilization of the specific structure of tRNAs [105, 341–343]. Enzymes accounting for pseudouridylation are known as PUS, and 13 such enzymes have been found in humans [338]. TruB pseudouridine synthase family member 1 (TRUB1) is a conserved mitochondrial Ψ synthase responsible for Ψ_55_ modification in mt-tRNA species, including mt-tRNA^Asn^, mt-tRNA^Gln^, mt-tRNA^Glu^, and mt-tRNA^Pro^ [344]. N1-specific methyltransferase modification of pseudouridine has been reported in archaea at position 54 in tRNA [345]. While Ψm has been detected in eukaryote, its functional role remains elusive [346].

##### Hydroxywybutosine (OHyW), Peroxywybutosine (o_2_yW)

Wybutosine (yW) is a heavily modified nucleoside present in tRNA^Phe^, and its derivatives, hydroxywybutosine (OHyW) and peroxywybutosine (o_2_yW), are located at position 37 in eukaryotes [347]. Derivatives of yW, which are critical for efficient codon recognition and the preservation of reading frames, are biosynthesized via a multistep reaction facilitated by TRMT5, tRNA Wybutosine-Synthesizing Protein 1 (TYW1), TYW2, TYW3, TYW5, and TYW4 [348, 349].

### Determinants of substrate specificity in tRNA modification enzymes

A fundamental question in the field of tRNA epitranscriptomics concerns the mechanisms by which modification enzymes attain precise recognition of their respective tRNAs within a milieu of structurally analogous substrates. Recent evidence suggests that this specificity is regulated through a multi-tiered mechanism. First, many tRNA modification enzymes function as heteromultimeric complexes, wherein a catalytically inactive subunit facilitates substrate binding and recognition. For instance, the m^1^A_58_ ‘writer’ TRMT6/TRMT61A necessitates TRMT6 for high-affinity tRNA binding, whereas the m^7^G_46_ ‘writer’ METTL1 relies on WDR4 for both stability and proper substrate positioning [350, 351]. Secondly, enzymes typically identify complex structural components rather than simple sequence motifs. The recognition of tRNA^Asp^ by DNMT2 requires a specific anticodon loop motif C^32^U^33^(G/I)^34^N^35^(C/U)^36^A^37^C^38^, in addition to structural characteristics such as the U11:A24 base pair within the D-stem and an appropriately sized variable loop [246]. Moreover, substrate selection can be dynamically regulated by the conformational state of the tRNA. The m^1^G_9_ methyltransferase Trm10 induces substrate-specific conformational changes in the tRNA core to gain access to the target nucleotide, a mechanism that distinguishes substrates from non-substrates despite their similar primary sequences [352]. Furthermore, the sequence of modifications establishes hierarchical specificity, wherein one modification functions as a prerequisite for the subsequent action of another enzyme. For instance, the initial m^1^G_37_ modification, catalyzed by TRM5, creates a particular chemical structure and recognition element, exemplifying hierarchical specificity. This design enables TYW1 to identify and act upon the modified substrate, thereby facilitating the next step in the catalytic cascade [349, 353]. Such layers of specificity ensure the accurate spatial and temporal deposition of tRNA modifications.

## The role of tRNA modifications in cancer development

**Fig. 5:**
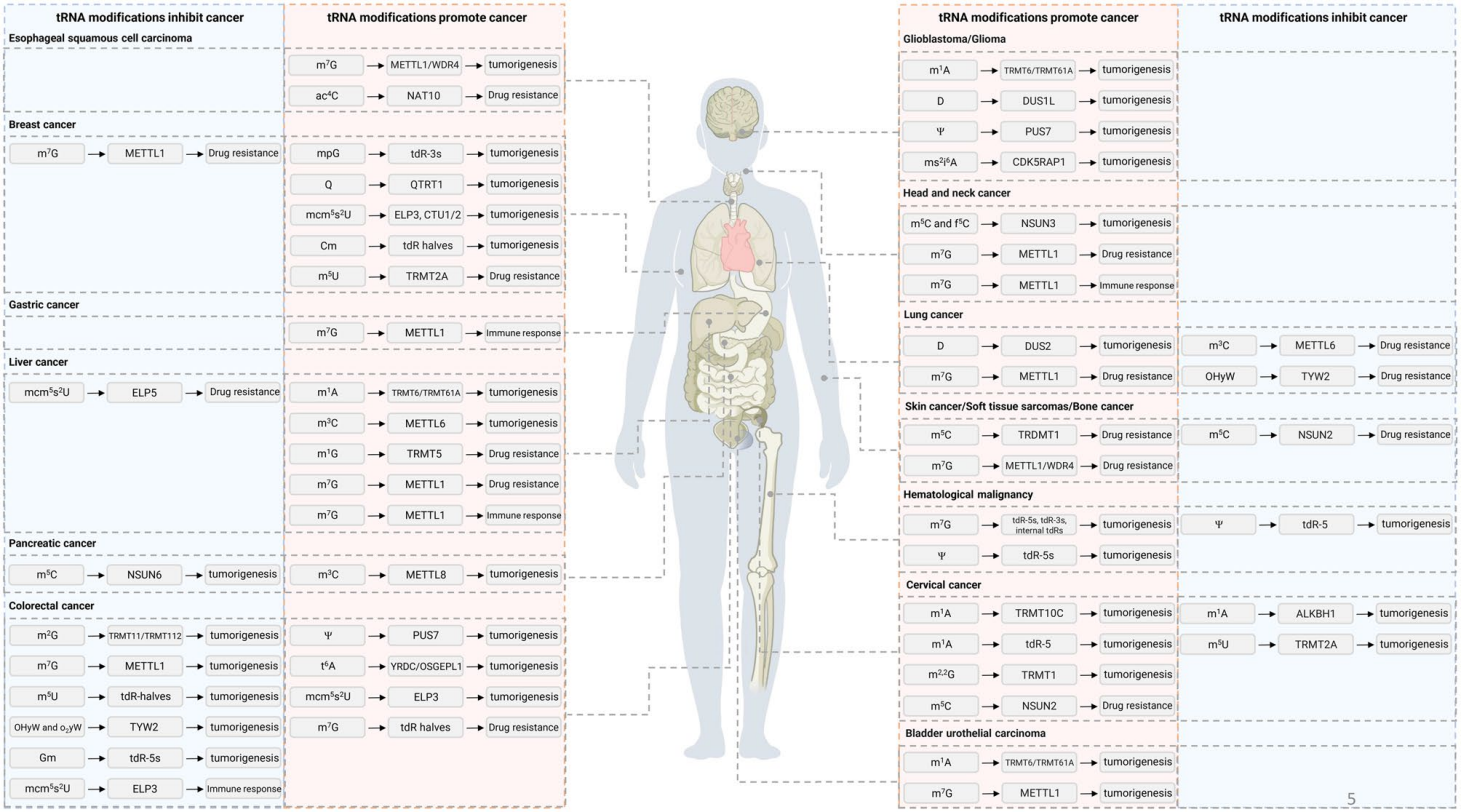
The role of tRNA modification in human cancer. Dysregulated tRNA modifications and their associated regulators, including ‘writers’, ‘erasers’, and ‘readers’, along with tRNA-derived RNAs in various human malignancies, and their roles in the promotion or suppression of tumorigenesis, migration/metastasis, drug resistance or immune response

**Fig. 6:**
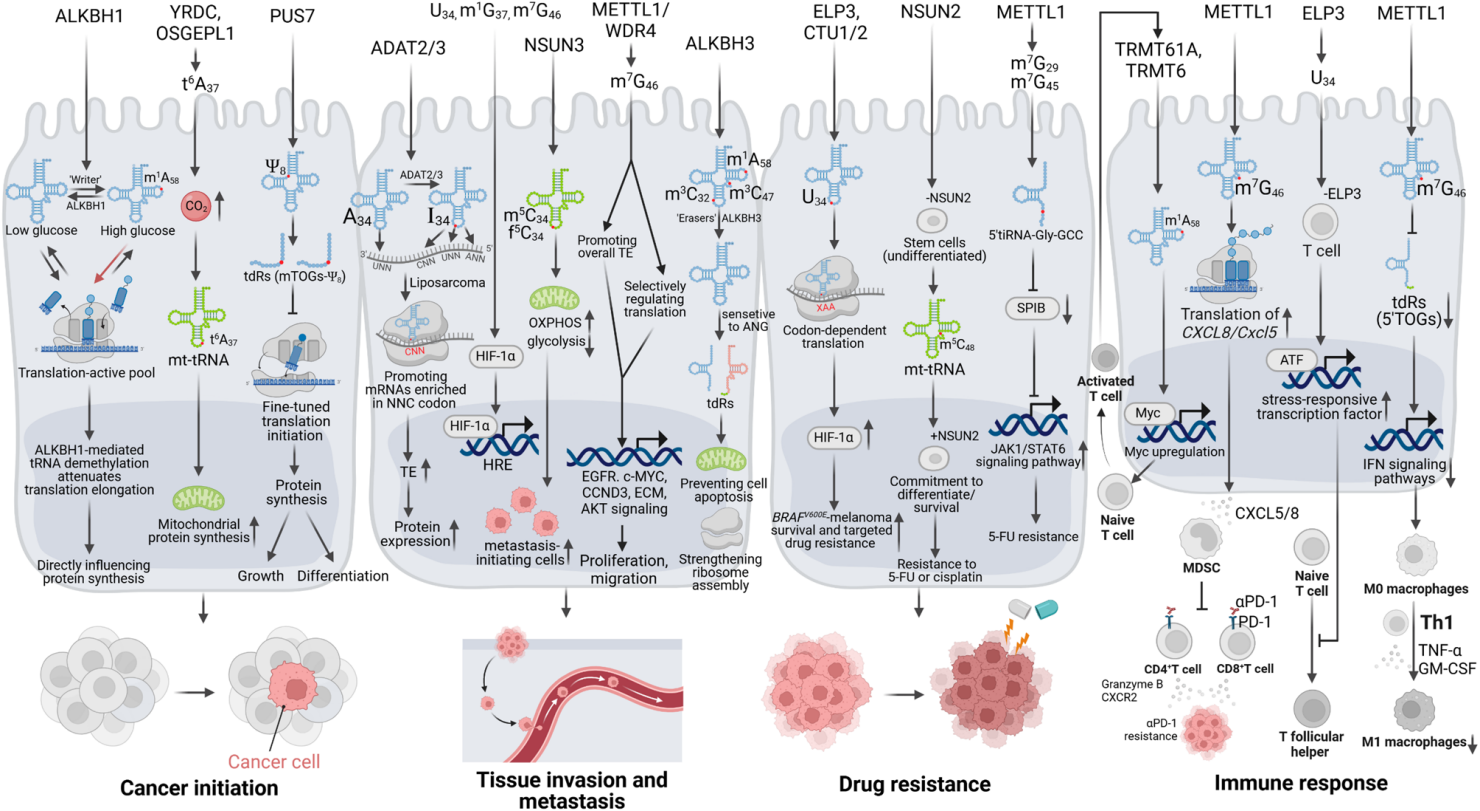
Dysregulation of tRNA modifications contributes to cancer development via diverse mechanisms. Aberrant transfer RNA (tRNA) modification epitomizes a hallmark of cancer that co-evolves with the dysregulation of ‘writers’, ‘erasers,’ and ‘readers’ (WERs), in conjunction with tRNA-derived RNAs (tdRs), collectively orchestrating tumor initiation, progression, drug resistance, and immune responses. Genetic alterations or environmental factors affecting tRNA modifiers could either enhance or attenuate their activity, therefore influencing the translation process and biosynthesis of tumor-associated proteins, as well as impacting oncogenic signaling, leading to the development of malignant phenotypes. These interactions culminate in augmented tumor cell proliferation, while simultaneously nurturing an immunosuppressive microenvironment that enables immune evasion, thereby facilitating initiation, progression and metastasis of cancer. 5-FU, 5-fluorouracil; ADAT2/3, adenosine deaminase tRNA specific 2/3; ALKBH1, AlkB homolog 1; ANG, angiogenin; CTU1/2, cytosolic thiouridylase subunits 1/2; ELP3, elongator complex protein 3; HIF-1α, hypoxia-inducible factor-1α; HRE, hypoxia response element; MDSC, myeloid-derived suppressor cell; METTL1, methyltransferase-like 1; mt-tRNA, mitochondrial tRNA; OSGEPL1, O-sialoglycoprotein endopeptidase like 1; OXPHOS, oxidative phosphorylation; PUS7, pseudouridine synthase 7; tdRs, tRNA-derived fragments; TE, translation efficiency; TRMT61A, tRNA methyltransferase 61A; WDR4, WD repeat domain 4; YRDC, yrdC N6-threonylcarbamoyltransferase domain containing

As the most abundant modification across all realms of life, tRNA modification is critically important, and its aberration regulation are well-documented phenomena that contribute to numerous facets of cancer development and progression. In this section, we will summarize the significance of tRNA modifications in a variety of cancers (Table 2; Fig. 5, 6).

**Table 2:** Summary of dysregulated tRNA modification in cancer

Cancer type	tRNA modification	Regulators	Distribution	Pathway involved	Mechanism of action	Level of evidence
*tRNA modifications promoting cancer*						
Acute myeloid leukemia	m^7^G	METTL1	tdR-5s, internal tdRs, and tdR-3s	NA	Knockdown of METTL1 reduces m^7^G modification on tRNA, destabilizing tRNAs and promoting tdRs biogenesis in AML cells	Cell lines
	Ψ	PUS7	tdR-5s	PABPC1	tdR-5s is regulated post-transcriptionally by PUS7-mediated pseudouridylation of U8, which directly affects mTOG–PABPC1 binding. This modulation influences the recruitment of PAIP1, a co-activator needed to stabilize interactions between PABPC1 and other eIF4F components	Cell lines and mouse models
Bladder urothelial carcinoma	m^1^A	TRMT6/TRMT61A	Cytoplasm	NA	The high expression of TRMT6/TRMT61A contributes to elevated m^1^A levels in urine and promotes the development of urinary bladder carcinoma	Cell lines
	m^1^A	TRMT6/TRMT61A	tdR-3b	UPR, CREB3L2, MBTPS1	TRMT6/61A promotes UPR by modifying tdR-3s and de-repressing their targets MBTPS1 and CREB3L2 in the ATF6 branch of UPR pathways	Cell lines
	m^7^G	METTL1	Cytoplasm	EGFR/EFEMP1	METTL1 controls translation of EGFR/EFEMP1 by modifying certain tRNAs	Cell lines and mouse models
	m^7^G	METTL1	tdR halves	ANXA2, Yes1	3'-tiRNA specifically binds ANXA2, promoting its Tyr24 phosphorylation by enhancing ANXA2-Yes1 interaction, leading to ANXA2 activation and increased nuclear localization of p-ANXA2–Y24 in BC cells	Cell lines and mouse models
Breast cancer	mpG	BCDIN3D	tdR-3s	miR-4454	BCDIN3D regulates the processing of tRNA^His^ 3'−fragments without impairing the canonical function of tRNA^His^ in aminoacylation	Cell lines
	Q	QTRT1	Cytoplasm	NA	Depletion of QTRT1 could suppress the proliferation and migration of BC cells	Cell lines and mouse models
	mcm^5^s^2^U	ELP3, CTU1/2	Cytoplasm	DEK, LEF1	ELP3 and CTU1/2 enhance invasion by translating oncoprotein DEK, which promotes IRES-dependent translation of proinvasive LEF1	Cell lines and mouse models
	Cm	TRMT13	tdR halves	NA	TRMT13-deficient BC cells accumulate 5’ tRNA-Gly-GCC halves, while TRMT13 promotes cell migration independently of its tRNA modification activity	Cell lines and mouse models
	m^5^U	TRMT2A	Cytoplasm	NA	HER2+/TRMT2A+ tumors in HER2-positive breast cancer patients show high recurrence risk when treated only with traditional cytotoxic therapies	Cell lines
Cervical cancer	m^1^A	TRMT10C	Mitochondria	NA	Silencing TRMT10C inhibits proliferation, colony formation, and migration in ovarian and cervical cancer cells	Cell lines
	m^1^A	ALKBH3	tdR-5	Cyt c	m^1^A-demethylated tRNA is more sensitive to ANG cleavage, producing conserved anticodon-region tdRs that enhance ribosome assembly and inhibit Cyt c-induced apoptosis	Cell lines
	m^2,2^G	TRMT1	Cytoplasm/ Mitochondria	NA	TRMT1-deficient cells exhibit reduced proliferation, impaired protein synthesis, disrupted redox homeostasis, elevated ROS, and heightened sensitivity to oxidants	Cell lines
	m^5^C	NSUN2	Mitochondria	NA	Double knockdown of NSUN2 and METTL1 potentiates sensitivity of cells to 5-FU whereas heat stress of cells shows no effects	Cell lines
Colorectal cancer	mcm^5^s^2^U	ELP3	Cytoplasm	Sox9	ELP3, induced by Wnt signaling, is essential for initiating colon cancer development by regulating Sox9 translation	Cell lines and mouse models
	t^6^A	YRDC/OSGEPL1	Mitochondria	Hypoxia	OSGEPL1 absence in cancer cells reduces growth by lowering oxygen consumption and decreasing complex I subunit protein levels in the electron transport chain	Cell lines and mouse models
	Ψ	PUS7	Cytoplasm	HSP90, LASP1	HSP90-dependent PUS7 upregulation enhances CRC metastasis by regulating LASP1	Cell lines and mouse models
	m^7^G	METTL1	tdR halves	JAK1/STAT6	5'tiRNA-Gly-GCC modulates the JAK1/STAT6 signaling pathway through SPIB targeting, while the synthesized poly (β-amino esters) enhances the delivery of both 5-FU and the 5’tiRNA-Gly-GCC inhibitor	Cell lines and mouse models
Esophageal squamous cell carcinoma	m^7^G	METTL1/WDR4	Cytoplasm	RPTOR/ULK1	Knockdown of METTL1 or WDR4 decreases m^7^G-modified tRNA levels and reduces translation of oncogenic transcripts in the RPTOR/ULK1/autophagy pathway	Cell lines and mouse models
	ac^4^C	NAT10	Cytoplasm	EGFR	NAT10 promotes esophageal cancer by enhancing EGFR translation efficiency, while its inhibition improves gefitinib treatment efficacy	Cell lines and mouse models
Gastric cancer	m^7^G	METTL1	NA	NA	GC patients in the low m^7^G-related gene group have increased immune cell infiltration, reduced immune escape/dysfunction, higher MSI-H, and improved immunotherapy efficacy	Bioinformatics
Glioblastoma	m^1^A	TRMT6/TRMT61A	Cytoplasm	PKCα	PKCα regulates TRM6/61 activity to prevent translation deregulation that may promote neoplastic development	Cell lines
	D	DUS1L	Cytoplasm	NA	DUS1L overexpression reduces UAC/UAU tyrosine codon translation and near-cognate UAA/UAG stop codon readthrough, likely due to decreased mature tRNA^Tyr^_GUA_ levels	Cell lines
	Ψ	PUS7	Cytoplasm	IFN	PUS7-mediated tRNA pseudouridylation is crucial for codon-specific translational control of glioblastoma stem cell regulators	Cell lines and mouse models
	ms^2^i^6^A	CDK5RAP1	Mitochondria	Autophagy	CDK5RAP1 abrogates the antitumor effect of i^6^A by converting it to ms^2^i^6^A, protecting glioma-initiating cells from excessive autophagy induced by i^6^A	Cell lines and mouse models
Head and neck cancer	m^7^G	METTL1	Cytoplasm	PI3K/AKT/mTOR	Deletion of METTL1 triggers the recruitment of CD4+ memory T cells, CD4+ naïve T cells, and CD8+ naïve T cells into the microenvironment	Cell lines and mouse models
	m^5^C and f^5^C	NSUN3	Mitochondria	CD36	CD36-dependent non-dividing, metastasis-initiating tumor cells need mitochondrial m^5^C to activate invasion and dissemination	Cell lines and mouse models
	m^7^G	METTL1	Cytoplasm	WNT/β-catenin, EMT	METTL1 upregulates WNT/β-catenin signaling, promoting NPC cell EMT and chemoresistance to cisplatin and docetaxel	Cell lines and mouse models
Liver cancer	m^1^A	TRMT6/TRMT61A	Cytoplasm	PPARδ	TRMT6/TRMT61A boosts m^1^A methylation in specific tRNAs, enhancing PPARδ translation, which promotes cholesterol synthesis, activates Hh signaling, and drives liver CSC self-renewal and tumorigenesis	Cell lines and mouse models
	m^3^C	METTL6	Cytoplasm	NA	Deletion of METTL6 alters ribosome occupancy and RNA levels, leading to impaired pluripotency	Cell lines and mouse models
	m^3^C	METTL6	Cytoplasm	NA	METTL6 regulates cell adhesion-related genes post-transcriptionally	Cell lines
	m^1^G	TRMT5	Mitochondria	HIF-1α	Knockdown of TRMT5 inactivates the HIF-1 signaling pathway by destabilizing HIF-1α through increased cellular oxygen levels and inhibiting TRMT5 sensitizes HCC to doxorubicin by modulating HIF-1α	Cell lines and mouse models
	m^7^G	METTL1	Cytoplasm	EGFR	m^7^G tRNA modification selectively controls the translation of oncogenic transcripts, including CCND3 and EGFR pathway genes, in codon-dependent mechanisms	Cell lines and mouse models
	m^7^G	METTL1	Cytoplasm	EGFR	METTL1 promotes translation of EGFR pathway genes to trigger Lenvatinib resistance	Cell lines and mouse models
	m^7^G	METTL1	Cytoplasm	CXCL8	METTL1 facilitates the accumulation of polymorphonuclear MDSCs by modulating the expression of CXCL8 via the regulation of tRNALys	Cell lines and mouse models
	m^7^G	METTL1	Cytoplasm	TGF-β2	Heat-induced upregulation of METTL1 enhances the translation of TGF-β2, thereby fostering an immunosuppressive milieu through the promotion of MDSC migration	Cell lines and mouse models
Lung cancer	D	DUS2	Cytoplasm	EPRS	DUS2 protein exhibits tRNA-DUS activity and physically interacts with EPRS, a glutamyl-prolyl tRNA synthetase, likely enhancing translational efficiency	Cell lines
	m^7^G	METTL1	Cytoplasm	AKT/mTORC1	METTL1 activates the AKT/mTORC1 signaling pathway	Cell lines
	m^7^G	METTL1	Cytoplasm	FOXM1, PTPN13	METTL1 enhances FOXM1 RNA stability and upregulates FOXM1, which transcriptionally suppresses PTPN13, reducing LUAD cell sensitivity to Gefitinib	Cell lines and mouse models
Melanoma	m^1^A	TRMT61B	Mitochondria	NA	TRMT61B depletion induces senescence in low-aneuploidy melanoma cells but triggers apoptosis in high-aneuploidy ones	Cell lines and mouse models
	mcm^5^s^2^U	ELP3 or CTU1 and/or CTU2	Cytoplasm	HIF-1α	U_34_ enzymes promote glycolysis in melanoma cells by directly regulating HIF1A mRNA translation and maintaining high HIF1α protein levels	Cell lines and mouse models
Neuroblastoma	m^7^G	METTL1	Cytoplasm	c-MYC	METTL1 knockdown reduces m^7^G tRNA modification, selectively suppressing oncogene mRNA translation in NBL in a codon frequency-dependent manner	Cell lines and mouse models
Pancreatic cancer	m^3^C	METTL8	Mitochondria	ND1/6	METTL8 depletion causes ribosome stalling on specific mt-tRNA^Ser^_UCN_ codons	Cell lines
Prostate cancer	m^7^G	METTL1	tdR-5	NA	m^7^G in tRNAs protects them from stress-induced cleavage and the formation of 5’ tRNA fragments	Cell lines and mouse models
	m^7^G	METTL1	tdR-5s	AKT/mTOR	METTL1 hinders the accumulation of tdR-5s, including 5'TOG derived from tRNA-Cys, in primary and metastatic prostate tumours	Cell lines and mouse models
Renal cancer	Ψ	PUS1	Cytoplasm	NA	Upregulated PUS1 increases RCC cell viability, migration, and invasion	Cell lines
	m^1^A	TRMT6	NA	NA	The rs236110 C > A SNP in TRMT6 is a Wilms tumor susceptibility locus	Bioinformatics
Skin cancer/Soft tissue sarcomas/bone cancer	m^7^G	METTL1/WDR4	Cytoplasm	ECM, LOXL2	METTL1/WDR4 modified tRNAs enhance the translation of mRNAs, including ECM remodeling effectors, facilitating osteosarcoma progression and chemoresistance to doxorubicin	Cell lines and mouse models
	m^5^C	TRDMT1	Cytoplasm	miR-23a-3p, miR-93-5p, miR-125a-5p, miR-191-5p	Knockout of TRDMT1 affects adaptive responses associated with protein homeostasis networks that during prolonged ER stress may result in increased sensitivity to apoptotic cell death	Cell lines
*tRNA modifications suppressing cancer*						
Acute lymphoblastic leukemia	Ψ	PUS7	tdR-5	PABPC1	Modification of uridine at position 8 by PUS7 in tRNA^Ala^, tRNA^Cys^, and tRNA^Val^ enhances tdR-5 biogenesis and promotes their binding to PABPC1, thereby modulating translation in human stem cells	Cell lines and mouse models
Breast cancer	m^7^G	METTL1	Cytoplasm	GADD45A, RB1	METTL1 amplifies the anti-tumor efficacy of abemaciclib and promotes the translation of GADD45A and RB1 mRNAs	Cell lines and mouse models
Cervical cancer	m^1^A	ALKBH1	Cytoplasm	NA	m^1^A demethylation impacts tRNAiMet levels and translation initiation, while ALKBH1-mediated tRNA demethylation reduces translation elongation	Cell lines
	m^5^U	TRMT2A	Cytoplasm	NA	TRMT2A overexpression enriches the G2/M phase population	Cell lines
Colorectal cancer	m^2^G	TRMT11/TRMT112	Cytoplasm	NA	m^2^Gs in the tRNA core function co-operatively to optimize protein synthesis for efficient cell proliferation	Cell lines
	m^5^U	NA	tdR-halves	NA	3'-tRNA-half mimic_M2 (m^5^U modified) enhances the stability of its tertiary structure and exhibits greater cytotoxicity than unmodified 3'-tRNA-half mimic	Cell lines
	m^7^G	METTL1	Cytoplasm	HIF-1α	tRNA m^7^G modification was down-regulated in CRC cells under hypoxia, dependent on HIF-1α-mediated inhibition of METTL1 transcription	Cell lines
	OHyW and o_2_yW	TYW2	Cytoplasm	ROBO1	Epigenetic loss of TYW2 leads to guanosine hypomodification in phenylalanine-tRNA, increased −1 frameshifts, and ROBO1 downregulation through mRNA decay	Cell lines
	Gm	NA	tdR-5s	NA	Gm modification of tRNA enhances the cytotoxic efficacy of tdR-5s from tRNA^Leu^_CAA_ in* E. coli* by stabilizing its tertiary structure, increasing its effectiveness against CRC cells	Cell lines and mouse models
	mcm^5^s^2^U	ELP3	Cytoplasm	mTORC2, Ric8b	ELP3 inhibits M1 but promotes M2 macrophage polarization by enhancing codon-dependent Ric8b translation	Cell lines and mouse models
Liver cancer	mcm^5^s^2^U	ELP5	Cytoplasm	hnRNPQ, P53	ELP5/hnRNPQ/P53 axis controls gemcitabine-induced cytotoxic effects in gallbladder cancer cells	Cell lines and mouse models
Lung cancer	m^3^C	METTL6	Cytoplasm	NA	Knockdown of METTL6, linked to rs2440915 SNP-correlated expression, significantly decreased cisplatin sensitivity in lung cancer cells	Cell lines
	OHyW	TYW2	Cytoplasm	NA	Low TYW2 expression promotes cancer survival and taxol resistance	Cell lines
Pancreatic cancer	m^5^C	NSUN6	Cytoplasm	NA	NSUN6 may affect pancreatic cancer cell growth by regulating CDK10, which is involved in mitotic spindle assembly and nuclear division	Cell lines and mouse models
Skin cancer/Soft tissue sarcomas/bone cancer	m^5^C	NSUN2	Mitochondria	Stress response	Inhibition of m^5^C locks tumor-initiating cells in a translational inhibition program linked to the stress response pathway	Cell lines and mouse models

^*^ Abbreviation NA: Not available

### Methylated tRNA modifications

m^1^A ‘writer’ TRMT6/TRMT61A complex is frequently up-regulated in cancers and it exerts protumorigenic function by promoting translation. In hepatocellular carcinoma (HCC), the upregulated TRMT6/TRMT61A elevates the m^1^A modification specifically in tRNA^Ala^_AGC_ and tRNA^Glu^_CTC_ to boost the translation of peroxisome proliferator-activated receptor delta (PPARδ), which in turn triggers cholesterol synthesis and activates Hedgehog signaling, ultimately driving self-renewal of cancer stem cells and tumorigenesis [354]. In aggressive glioblastoma multiforme TRMT6/TRMT61A mediates tRNA^Met^ m^1^A modification to drive the translation of a subset of mRNAs involved in tumorigenesis [198]. In bladder cancer, elevated expression of TRMT6/TRMT61A increases urinary m^1^A and promotes malignant phenotypes [197]. In connection with experimental work, single nucleotide polymorphism (SNP) in *TRMT6* gene is associated with an increased susceptibility to hepatoblastoma and Wilms tumor in pediatric populations, and in both studies rs236110 C > A was identified to be a susceptibility locus [355, 356]. More studies are required to elucidate functionality of potentially pathogenic TRMT6 variants in tumorigenesis. Meanwhile, m^1^A and m^3^C ‘eraser’ ALKBH3 is also important for cell proliferation and cell migration in various cancer cell lines, as well as for tumor progression in xenograft models [178, 200]. Mechanistically, tRNA with demethylated m^1^A exhibits increased sensitivity to angiogenin cleavage, resulting in generation of tdRs that potentiate translation by recruiting the small subunit of ribosomes and interact with cytochrome *c* (Cyt *c*) to impair apoptotic processes [178]. In contrast, m^1^A ‘eraser’ ALKBH1 has been reported to function as a tumor suppressor. ALKBH1-driven m^1^A demethylation of tRNA^Met^ attenuated protein translation, and its knockdown in HeLa cells promotes translation and proliferation [201]. The m^1^A modification of tRNA not only regulates the biological functions of tRNAs but also exerts a detrimental effect on gene silencing mediated by tdRs [168]. In bladder cancer, the overexpression of TRMT6/TRMT61A introduces m^1^A_58_ into the seed region of tdR-3b, disrupting its Watson-Crick base pairing with target mRNAs such as CAMP-responsive element-binding protein 3 like 2 (*CREB3L2*) and membrane-bound transcription factor peptidase, site 1 (*MBTPS1*), which subsequently promotes a pro-survival unfolded protein response and potentiates cell proliferation [168].

METTL6 is implicated in m^3^C methylation of cyto-tRNA^Ser^_GCU_ and is commonly regarded as an oncogene [235]. *METTL6* is amplified in patients with highly proliferative luminal breast cancer, and its expression correlates with poor prognosis [357]. Notably, deletion of *METTL6* reduces m^3^C levels on tRNA^Ser^, resulting in decreased ribosome occupancy on mRNAs encoding proliferation-related proteins and dampened HCC tumorigenesis [358]. METTL6 also sustains the invasive phenotype of HCC cells by modulating the expression of genes associated with cell adhesion [359]. METTL2A, recognized as a ‘writer’ for m^3^C_32_ modifications on tRNA^Thr^ and tRNA^Ser^, was found to be upregulated and to correlate with an unfavorable outcome in breast invasive carcinoma [360]. In pancreatic cancer, the levels of NSUN6 are diminished, and its tumor suppressive role has been validated using in vitro and in vivo functional assays [361]. Mechanistic evidence indicates that NSUN6 restricts pancreatic cancer cell growth by promoting the expression of cyclin-dependent kinase 10 (CDK10), a tumor suppressor implicated in mitotic spindle assembly and nuclear division during mitosis [361]. m^5^C modifications driven by NSUN2 contribute to stabilization of tRNA secondary structures, thereby safeguarding them against cleavage by angiogenin [177]. Interestingly, deletion of *NSUN2* enhances the self-renewal capacity of tumor-initiating cells (TICs) by maintaining low translation rates, leading to an expanded cell population that contributes to skin carcinogenesis characterized by poorly differentiated, high-grade features [362]. In contrast, in anaplastic thyroid cancer, elevated expression of NSUN2 promotes tumor progression primarily through m^5^C modification of tRNA^Leu^ [247]. This stabilizes tRNA^Leu^ and enhances protein translation efficiency, which is essential for supporting a pro-cancer translational program and contributing to a codon-dependent translation bias [247].

METTL1/WDR4 complex, which catalyzes tRNA m^7^G_46_ modification, is overexpressed in many cancers and primarily functions as an oncogene [363]. The elevated tRNA m^7^G_46_ modification mediated by METTL1/WDR4 in cancer not only promotes overall translation efficiency but also enables m^7^G-modified tRNAs to decode a greater array of codons in mRNAs characterized by diminished translation rates, whereas reduced m^7^G modification on tRNAs impairs mRNA translation by triggering ribosomal stalling at codons decoded by m^7^G-modified tRNAs [320, 364–368]. These mechanisms allow selective translation of mRNA associated with tumor-related genes and pathways such as *c-MYC* [364], *cyclin D3* [365], epidermal growth factor receptor (*EGFR*) [366, 367], phosphatidylinositol 3-kinase (*PI3K*), protein kinase B (*AKT*), and extracellular matrix (ECM) remodeling pathways [36, 368], thereby promoting tumorigenesis and metastasis. METTL1 regulates stress response in cancer cells. In lung cancer cells, METTL1 represses autophagy by activating of AKT and mammalian target of rapamycin complex 1 (mTORC1) signaling pathway [369]. *METTL1* deletion in prostate cancer (PCa) impairs the resolution of autophagy and exacerbates proteostatic stress, leading to the accumulation of reactive oxygen species (ROS) and DNA damage, leading to impaired tumor growth [370]. Inverse correlation between METTL1 and autophagy was validated in esophageal squamous cell carcinoma [371]. Mechanistically, METTL1-mediated m^7^G modification of tRNAs protect them from stress-induced damage [370]. Furthermore, METTL1-dependent translation of regulatory-associated protein of mTOR complex 1 (*RPTOR*) activates mTOR to negatively regulate autophagy [371]. On the other hand, hypoxia negatively regulates *METTL1* via binding of hypoxia-inducible factor-1α (HIF-1α) to hypoxia response element in *METTL1* promoter. This concomitantly down-regulated METTL1 and m^7^G modification, implying a negative feed-back loop that enables adaptation to environmental stressors [372]. METTL1 also promotes tumorigenesis via its impact on tdRs. In leukemia, *METTL1* loss reduces m^7^G modifications on tRNAs, which destabilizes tRNAs and promotes biogenesis of tdRs, including tdR-5s, internal tdRs, and tdR-3s, which impede leukemogenesis [373]. A recent study identified a novel tdR, 3'-tiRNA^Lys^_TTT_ (mtiRL), mediated by METTL1 that enhances the malignancy of bladder cancer by specifically binding to the oncoprotein Annexin A2 (ANXA2), and facilitating ANXA2 phosphorylation at Tyr24 via the increased interactions with Yes proto-oncogene 1 (Yes1), thereby activating ANXA2 and promoting its nuclear localization in bladder cancer cells [374]. TRMT5, the enzyme responsible for m^1^G_37_ tRNA modification, is upregulated in HCC and associated with a poor prognosis [375]. *TRMT5* knockdown inactivates HIF-1 signaling pathway by enhancing cellular oxygen levels, which destabilizes HIF-1α and ultimately attenuates the proliferation and metastasis of HCC [375]. Downregulation of tdR-3s of tRNA^His^ was identified in breast cancer cells due to elevated expression of BCDIN3D, an RNA phospho-methyltransferase responsible for methylating the 5’ monophosphate of specific RNA molecules [376, 377].

The impact of m^2^ G and m^2,2^G regulators on tumorigenesis is a subject of ongoing debate [305, 309, 378, 379]. Knockdown of putative methyltransferases, *THUMPD3* or *TRMT11*, conferred differential phenotypic outcomes in colorectal cancer (CRC) cells, whereas simultaneous knockout of both suppressed protein synthesis and cell proliferation [305]. Hence, further validation is essential to assess the function of tRNA m^2^ G and m^2,2^G modifications in tumorigenesis of diverse cancer cell types.

### Mitochondrial tRNA modifications

Beyond cytosolic tRNAs, TRMT61B-driven m^1^A_58_ modification in mt-tRNAs is positively correlated with aneuploidy in human cancers [380]. In cancer cells with high aneuploidy, TRMT61B is pivotal in modulating mitochondrial function. *TRMT61B* depletion reduced levels of electron transport chain components, leading to attenuated mitochondrial function and cell death, suggesting that TRMT61B might be a potential target for tumors with high aneuploidy [380]. Increased expression of TRMT10C, which mediates m^1^A_9_ methylation in mt-tRNA, has been linked to unfavorable prognosis in cervical carcinoma, endometrial carcinoma, ovarian cancer, and HCC [140, 381]. Its oncogenic function has been validated by in vitro studies involving ovarian and cervical cancer cells [140].

Another m^3^C methyltransferase, METTL8, is essential for mitochondrial function through catalyzing methylation of mt-tRNA^Ser^_UCN_ and mt-tRNA^Thr^, thereby orchestrating metabolic reprogramming of cancer cells [238, 382]. In aggressive pancreatic cancer cells, *METTL8* deletion suppresses m^3^C levels on mt-tRNA, resulting in ribosomal stalling at codons for mt-tRNA^Ser^_UCN_, ultimately impairing function of mitochondrial respiratory chain and cell survival [238]. Delaunay et al. [40] demonstrate that NSUN3-dependent m^5^C modification of mt-tRNA^Met^ and further metabolism into f^5^C_34_ via ALKBH5 is essential for translation of mitochondrial mRNA, thus boosting metabolic plasticity via oxidative phosphorylation. In this regard, CD36-dependent tumor cells that initiate metastasis cascade require NSUN3-induced m^5^C modification to effectively trigger invasion and dissemination [40].

Mitochondrial methyl-thio-modifying enzyme CDK5RAP1, which catalyzes conversion of i^6^A to ms^2^i^6^A at position A_37_ in mt-tRNAs, is integral to sustaining tumor-propagating and self-renewal capacities of glioblastoma (GBM) stem cells [141]. Although its deficiency does not impair mitochondrial respiration, it leads to the loss of certain electron transport chain components, altering mitochondrial metabolic phenotype and enhancing autophagy, a mechanism that cells exploit during gliomagenesis to develop a malignant phenotype [141]. YRDC and O-sialoglycoprotein endopeptidase like 1 (OSGEPL1) are required for the t^6^A modification at position 37 of mt-tRNAs, including mt-tRNA^Ser^, mt-tRNA^Thr^, mt-tRNA^Asn^, mt-tRNA^Ile^, and mt-tRNA^Lys^ [383]. This modification is implicated in adaptation of cancer cells to high CO_2_/bicarbonate concentrations present in hypoxia conditions in solid tumors. Depletion of *OSGEPL1* in HeLa cells exhibits reduced growth attributed to a lower oxygen consumption rate (OCR) and reduced protein levels of subunits of complex I of the electron transport chain [59].

### Wobble modifications

An increasing amount of evidence implicates modifications within the anticodon loop, particularly at the wobble position, in the fine-tuning of oncogene expression. These modifications impact codon-biased translation of critical cancer-related transcripts and are often dysregulated in malignancies. A recent publication reported that ADAT2-mediated tRNA A-to-I modifications at the wobble position promote colorectal tumorigenesis through the HDAC7-WNT/β-catenin signaling pathway. ADAT2 expression functions as an independent prognostic indicator, correlating with unfavorable outcomes in CRC patients [384]. Furthermore, Ramirez-Moya et al. [385] showed that the genes encoding the ADAT2/3 deaminase complex are frequently amplified and/or overexpressed in various tumor types, including liposarcoma.

In CRC, the mcm^5^s^2^U modification enzyme ELP3 plays an important role in the initiation of WNT-driven intestinal tumors [386]. Upregulation of ELP3, induced by WNT signaling, enhances translation of SRY-Box transcription factor 9 (SOX9), which is notably enriched with mcm^5^s^2^U-sensitive codon AAA, and ELP3-mediated SOX9 up-regulation is essential for the maintenance of intestinal cancer stem cells [386]. In breast cancer, ELP3 enhances the translation of DEK protooncogene, which in turn promotes IRES-mediated translation of LEF1 and facilitates tumor cell invasion and metastasis [387]. tRNA queuosine modification enzyme QTRT1 regulates genes critical for cell proliferation, tight junction formation, and migration, and *QTRT1* knockdown impedes the proliferation of human breast cancer cells in vitro and in vivo [388].

### Other modifications

PUS7 mediates U_50_ pseudouridylation in tRNAs, which is associated with oncogenesis in GBM. In this regard, the principal pro-tumorigenic function of PUS7 has been attributed to the pseudouridylation of tRNA^Arg^_CCG_, which in turn promotes the growth of glioma stem cells via the tyrosine kinase 2 (TYK2)-mediated interferon (IFN) pathway [35]. In CRC and renal cell carcinoma, PUS7 correlates with advanced clinical stage and poor survival, and functionally promotes pro-metastatic phenotypes [389, 390], however, such effects are independent of Ψ catalysis [389]. PUS10 catalyzes Ψ_54_ and Ψ_55_ formation in a subset of tRNAs, with its pseudouridylation activity proving essential for tumor cell proliferation [391]. Concurrently, PUS10 enhances the biogenesis of diverse microRNAs, a process that occurs independently of its Ψ synthase activity [391]. On the other hand, PUS7 has been shown to function as a tumor suppressor in other settings. In myelodysplastic syndrome (MDS), PUS7-driven Ψ modification in a stem cell-enriched tdR subtype 2, mini tdRs featuring a 5’ terminal oligoguanine (mTOG), selectively inhibits aberrant protein synthesis to facilitate the engraftment and differentiation of hematopoietic stem and progenitor cells (HSPCs) in patients with MDS. Hence, dysregulation of PUS7-mTOG aberrantly elevates translation of transcripts with 5’ pyrimidine-enriched sequences (PES), and is correlated with leukemic transformation and reduced patient survival [179]. The introduction of Ψ at the U_8_ position of tRNAs by PUS7 also suppresses tumorigenesis, through the activation of a network of tdRs that specifically target the translation initiation complex [55]. Inactivation of PUS7 thus disrupts tdRs-mediated translational regulation, leading to elevated protein biosynthesis and impaired germ layer specification, hampering hematopoietic stem cell commitment and promoting aggressive subtypes of MDS [55]. DUS1L, the ‘writer’ responsible for dihydrouridine modification in tRNA D_16_/D_17_, is over-expressed in glioma patients with poor prognosis [392]. In this regard, the overexpression of DUS1L leads to a reduction in tRNA^Tyr^_GUA_, subsequently diminishing the mistranslation of UAA and UAG codons [392]. DUS2, another D ‘writer’, physically interacts with EPRS, a glutamyl-prolyl tRNA synthetase, potentially enhancing translational efficiencies, and it is frequently upregulated in non-small cell lung cancer [393].

As a modified nucleoside derived from the turnover of i^6^A-modified tRNA, i^6^A is present in mammalian cells, excreted in urine, and has been studied since the late 1960s for its inhibitory effects on cellular replication in various cancer cell lines [394–397]. However, in vivo results demonstrate that it is ineffective as an anticancer agent, attributed to its rapid metabolism [398–404]. In CRC, the epigenetic silencing of *TYW2* triggers hypomodification of OHyW and o_2_yW at position 37 in tRNA^Phe^, leading to an increase in − 1 ribosomal frameshift events and the downregulation of transcripts, including tumor suppressor *ROBO1*, via nonsense-mediated mRNA decay [405]. TYW2 loss therefore augments migratory capabilities and epithelial-to-mesenchymal transition characteristics in CRC cells [405].

### Microbial tRNA modification and cancer

The gut microbiota may generate tdRs that arise from tRNA modifications, which has been reported to modulate tumor development [406]. Specifically, investigators have discovered that the Gm modification of tRNA may enhance the cytotoxic effect of tdR-5s derived from tRNA^Leu^_CAA_ in *Escherichia coli* by augmenting the stability of its tertiary structure, thereby increasing its efficacy against CRC cells [406]. Modification of m^5^U on tRNA^Ile^ in fungus *Ganoderma lucidum* has been shown to enhance cytotoxicity against cancer cells, an effect due to increased stability of tertiary structure of the 3'-t-half mimic derived from tRNA^Ile^ [407]. These studies suggest modified microbial tRNAs as potential anticancer agents.

### tRNA modifications in cancer therapy response

tRNA-modifying enzymes not only drive tumorigenesis but also critically modulate the response of cancer cells to genotoxic stress and targeted agents. The following section summarizes how specific modifications and their regulators influence sensitivity or resistance to chemotherapy, radiotherapy, and targeted therapies.

A genome-wide association study (GWAS) for cisplatin cytotoxicity showed that *METTL6* expression is associated with rs2440915 SNP. *METTL6* knockdown significantly reduced cisplatin sensitivity in lung cancer cells [408], but whether it is a consequence of m^3^C tRNA modification remains unclear. The loss of DNMT2, which is involved in the m^5^C_38_ modification of tRNA^Asp^ and has been demonstrated to enhance sensitivity to DNA double-strand breaks (DSBs), suggests that its inhibition may represent a promising strategy in conjunction with DNA damage repair (DDR) inhibitors [409]. DNMT2-mediated mRNA m^5^C modification at DNA damage sites promotes recruitment of RAD51 recombinase (RAD51) and RAD52 and enhancing DNA damage repair through homologous recombination [410]. The absence of DNMT2 in osteosarcoma cells not only increases their sensitivity to DSBs, but also confers poly(ADP-ribose) polymerase (PARP) inhibitor sensitivity [410]. Moreover, knockout of *DNMT2* influences the unfolded protein response, enhancing cellular vulnerability to apoptosis triggered by endoplasmic reticulum stress (ERS) subsequent to doxorubicin administration [411]. m^5^C modifications in tRNA driven by NSUN2 has been linked to drug resistance in cancer. In human cutaneous tumors, NSUN2-deficient TICs demonstrate increased sensitivity to treatment with 5-Fluorouracil (5-FU) or cisplatin, attributable to the failure to activate pro-survival pathways in response to stress [362]. In HeLa cells, collaborative action of NSUN2 and METTL1 protects from 5-FU-induced cytotoxicity by preserving tRNA stability, while having no effect on response to paclitaxel and cisplatin. These results suggest that inhibitors of NSUN2 might be effective in enhancing the efficacy of select chemotherapeutic agents [412].

The inhibition of TRMT5 potentiates the sensitivity of HCC to doxorubicin by modulating HIF-1α levels [375]. In HCC, METTL1 promotes tumor resistance to radiotherapy by enhancing the translation of the DNA double-strand breaks repair enzyme DNA ligase IV, while the upregulation of m^7^G has also been demonstrated to enhance translation of SLUG and SNAIL, thereby promoting epithelial-to-mesenchymal transition and reinstating malignant potential following inadequate radio-frequency ablation [413, 414]. Additionally, the depletion of *METTL1* in PCa increased sensitivity to chemotherapy, including docetaxel and etoposide, and is also associated with chemosensitivity of nasopharyngeal carcinoma cells in response to cisplatin and docetaxel [370, 415]. Furthermore, elevated METTL1 expression has been correlated with resistance to doxorubicin in osteosarcoma, a phenomenon mediated by increased translational activity of Lysyl oxidase-like 2 (LOXL2), an oncogenic enzyme that facilitates the crosslinking of elastin and collagen within the ECM [368]. The specific 5'half tRNA, 5'tiRNA-Gly-GCC, which is upregulated in CRC and m^7^G-modified by METTL1, was reported to promote 5-FU resistance via JAK1/STAT6/SPIB signaling pathway [416]. Hence, targeting METTL1 or associated m^7^G-modified tRNAs may promote radiotherapy and chemotherapy efficacy. Furthermore, it has been supported that m^7^G tRNA modification is crucial for augmenting resistance to Lenvatinib and Gefitinib, targeted cancer drugs classified as tyrosine kinase inhibitors (TKIs) [417, 418]. On the contrary, overexpression of METTL1 amplifies the antitumor efficacy of Abemaciclib, an inhibitor of cyclin-dependent kinases 4/6 (CDK4/6) [419]. Upregulation of m^2^ G and m^2,2^G modifications, mediated by TRMT1, contribute to radio-resistance and metastatic transformation in breast cancer by enhancing proliferation rates, modulating global protein synthesis, and maintaining redox homeostasis [420, 421].

In patients with human epidermal growth factor receptor 2 (HER2)-positive breast cancer, positivity for TRMT2A predicts a high recurrence risk when treated solely with traditional cytotoxic therapies [422]. Hence, a more aggressive treatment strategy in adjuvant setting should be considered for these patients independent of other conventional risk factors [422]. Enzymes catalyzing tRNA modifications of uridine at the wobble position (U_34_ enzymes) have been implicated in resistance to targeted therapies. Notably, ELP3 and CTU1/2 have been demonstrated to promote glycolysis in BRAF^V600E^ therapy-resistant melanoma cells by up-regulating translation of HIF-1α through codon-biased translation of uridine (U_34_)-modified tRNAs [423]. U_34_ enzymes thus contribute to therapy resistance against RAF inhibitors, including vemurafenib and dabrafenib [423]. In another study, *ELP5* is identified to be required for response to gemcitabine in gallbladder cancer by leveraging genome-wide CRISPR screening [424]. Loss of ELP5 leads to U_34_ hypomodification and dampened the translation of heterogeneous nuclear ribonucleoprotein Q (hnRNPQ), which inhibits TP53 IRES-dependent translation, ultimately decreasing sensitivity of gallbladder cancer cells to gemcitabine [424].

NAT10 enhances tRNA stability via ac^4^C modification and mediates resistance to targeted therapy in esophageal cancer by augmenting the translation efficiency of EGFR. Therefore, NAT10 blockade markedly improves the effectiveness of an anti-EGFR inhibitor (Gefitinib) in this context [425]. Profiling of tRNA modification in human cancer cells and their taxol-resistant counterparts identified the substitution of OHyW by 4-demethylwyosine at the 37th position of tRNA^Phe^, which correlates with TYW2 downregulation. *TYW2* knockdown causes the accumulation of 4-demethylwyosine and enhances resistance to taxol therapy [125], representing a novel mechanism of taxol resistance.

### tRNA modifications in antitumor immunity

Beyond cell-autonomous effects, emerging studies show that tRNA modifications modulate the TME and antitumor immunity by controlling the translation of key immune regulators in cancer and immune cells, thereby influencing immune cell function. This identifies tRNA modifiers as potential targets to enhance immunotherapy efficacy.

In intrahepatic cholangiocarcinoma, METTL1 promotes enrichment of polymorphonuclear myeloid-derived suppressor cells (MDSCs) by modulating the expression of *CXCL8* via regulation of tRNA^Lys^ [34]. Consequently, co-blocking of METTL1 and CXCL8–CXCR2 axis promoted anti-PD1 efficacy in mouse tumor models [34]. Similarly, radiofrequency ablation-induced up-regulation of METTL1 in HCC enhances translation of transforming growth factor β2 (TGF-β2), thereby fostering an immunosuppressive milieu by promoting MDSC migration [426]. Converse to its effect on MDSCs, *METTL1* deletion triggers the recruitment of CD4^+^ memory T cells, CD4^+^ naïve T cells, and CD8^+^ naïve T cells into the microenvironment of head and neck squamous cell carcinoma [36]. Consistent with these experimental findings, gastric cancer patients with low m^7^G-related gene activities exhibit increased immune cell infiltration and tumor mutation burden, as well as enhanced efficacy towards immunotherapy [427]. Furthermore, the depletion of METTL1-mediated m^7^G tRNA results in the generation of a novel class of tdRs characterized by a 5’-oligoguanine domain (5'TOG) in PCa, with these tdR-5s adeptly directing the translation of regulators of IFN pathway and immune effectors [428]. This in turn leads to the increased infiltration of pro-inflammatory immune cells and enhanced immunotherapy response.

Translation reprogramming, which occurs during macrophage polarization, requires tRNA modifications [429]. The U_34_ enzyme ELP3 is instrumental in this process as it restricts M1 macrophage polarization whilst preferentially enhancing pro-tumoral M2 macrophage by facilitating the codon-dependent translation of RIC8B, an essential regulator of mTORC2 [429]. While ELP3-deficient T cells exhibit diminished expansion, leading to compromised functionality in T follicular helper (TFH) responses [430].

## tRNA modification detection and cancer diagnostic implications

The clinical translation of tRNA modification research depends critically on robust methods for its detection and quantification. Over the past decade, technological advances have enabled increasingly sensitive and comprehensive profiling of tRNA modifications in both tissue samples and biofluids. These tools are now paving the way for the development of tRNA modification-based biomarkers for cancer diagnosis, prognosis, and therapeutic monitoring.

### Technological advances in detecting tRNA modifications

Current approaches for tRNA modification analysis can be broadly categorized into mass spectrometry (MS)-based, next-generation sequencing (NGS)-based, and third-generation sequencing (nanopore)-based approaches. MS-based methods, particularly liquid chromatography-tandem mass spectrometry (LC-MS/MS), offer enhanced sensitivity and accuracy for quantifying both known and novel modifications. The principle relies on resolving digested nucleosides by liquid chromatography and analyzing their mass-to-charge ratios and fragmentation patterns [431–435]. Advances such as higher-energy collisional dissociation (HCD) and ion mobility enable discrimination of positional isomers [436, 437]. However, MS methods require specialized expertise, relatively large amounts of input material, and are not readily amenable to high-throughput analysis [438, 439]. For clinical translation, key challenges include standardizing sample preparation, ensuring batch-to-batch reproducibility, and developing robust internal standards for absolute quantification.

NGS-based technologies have emerged as highly sensitive tools for mapping tRNA modifications at single-base resolution. These methods can be classified into three main strategies: direct sequencing, chemical-derivatization and sequencing, and antibody-pulldown and sequencing [440]. Direct sequencing techniques, such as DM-tRNA-seq, ARM-seq, and mim-tRNA-seq, are developed for the general purpose of tRNA sequencing and are adept at mapping specific modifications in which methylation-dependent mutation signals are detected by sequencing [441–443]. ARM-seq tackles the problem of reverse transcriptase (RT) stalling at modified sites by pre-treating RNA with *E. coli* AlkB demethylase to remove some common ‘hard-stop’ modifications [441]. mim-tRNA-seq adopts an opposite strategy. Instead of removing modifications, it optimizes conditions for the thermostable group II reverse transcriptase (TGIRT) to read through them. This process generates misincorporation signatures at modified sites, allowing simultaneous quantification of tRNA abundance, aminoacylation, and modification status in a single experiment [443]. Chemical derivatization represents a more sophisticated approach for distinguishing the modified nucleotides from their unmodified counterparts [444]. Antibody-based pulldown strategies have also been developed to selectively enrich and map RNA modifications, a process that involves fragmenting isolated RNA and using specific antibodies for immunoprecipitation to enrich modification-containing fragments [445–447]. While NGS methods enable genome-wide profiling from limited samples, they are often modification-specific, susceptible to RT bias, and face challenges in detecting multiple modifications on the same tRNA molecule or distinguishing closely related modifications [448]. Clinical adoption requires rigorous validation against orthogonal methods, establishment of standardized bioinformatics pipelines, and demonstration of reproducibility across laboratories.

Recently, a promising alternative to NGS-based technologies for characterizing the tRNAome is the direct RNA sequencing (DRS) platform developed by Oxford Nanopore Technologies [449, 450]. By threading native RNA molecules through a protein nanopore, it directly measures ionic current shifts that reflect both the canonical base and its modifications. This innovative technology enables the direct sequencing of native RNA molecules, allowing for the detection and quantification of both tRNA modifications and abundances without the necessity of RT or additional polymerase chain reaction [451–456]. However, challenges remain in basecalling algorithms for modified bases, throughput, and cost. For clinical deployment, rigorous quality control and harmonization of analysis pipelines are essential. (Summarized in Table 3).

**Table 3:** Overview of tRNA modification detection technologies

Technology category	Specific method(s)	Key principle	Detected modifications (examples)	Advantages	Limitations
**Mass Spectrometry (MS)-based**	LC-MS/MS, HILIC-MS/MS	Quantitative and qualitative analysis of nucleoside masses after hydrolysis	m^1^A, m^3^C, m^1^G, m^2,2^G, m^5^C, Ψ	High accuracy and sensitivity; Gold standard for quantification	Low-throughput; Requires specialized expertise; Cannot provide sequence context
**Next-Generation Sequencing (NGS)-based**					
Direct sequencing	DM-tRNA-seq, ARM-seq, mim-tRNA-seq	Detects reverse transcription (RT) stops, drops, or mutations caused by modifications	m^1^A, m^3^C, m^1^G, m^2,2^G	Maps modifications to specific tRNA sequences; High-throughput	Complex data analysis; Can miss low-abundance modifications
Chemical derivatization	Ψ-seq, PSI-seq	Chemical tagging of modifications to induce specific RT signatures	Ψ	High-resolution, specific mapping of targeted modifications	Requires optimization for each modification; Complex sample preparation
	RBS-seq	Bisulfite treatment to deaminate unmodified C, leaving m^5^C unchanged	m^5^C	Specific detection of m^5^C; Adaptable from DNA bisulfite sequencing	Harsh chemical treatment can degrade RNA
	AlkAniline-seq, TRAC-seq	Alkaline hydrolysis and aniline cleavage at abasic sites	m^7^G, m^3^C	Pinpoints modification sites at single-nucleotide resolution	Complex multi-step protocol
	Rho-seq	Chemical derivatization with NaBH4 and rhodamine	D (Dihydrouridine)	Specific method for detecting D modification	Specialized protocol for a single modification
Antibody pulldown	m^7^G-MeRIP-seq	Immunoprecipitation of modified RNA fragments with specific antibodies	m^7^G, m^6^A, m^5^C	Enriches for low-abundance transcripts; Well-established protocol	Antibody specificity issues; Lower resolution (~100–200 nt)
**Third-generation sequencing**	Nanopore DRS (Nano-tRNAseq)	Direct sequencing of RNA by measuring current changes as strands pass through a nanopore	Multiple modifications simultaneously	Detects abundance and modifications simultaneously; No RT or PCR bias; Long reads	High error rate in base calling; Requires specialized algorithms for modification calling

^*^ Abbreviation DRS, direct RNA sequencing; HILIC-MS/MS, hydrophilic interaction liquid chromatography-tandem mass spectrometry; HPLC, high-performance liquid chromatography; LC-MS/MS, liquid chromatography-tandem mass spectrometry; RT, reverse transcription

### Clinical implications of tRNA modification signatures in cancer

The dysregulation of tRNA modifications in cancer, coupled with the release of modified nucleosides and tdRs into biofluids, offers unique opportunities for non-invasive cancer detection and monitoring.

#### Modified nucleosides as liquid biopsy biomarkers

Modified nucleosides derived from tRNA are excreted in urine and can be quantified by MS-based methods, providing a non-invasive window into tumor-associated epitranscriptomic alterations. For instance, an ultra-high-performance LC-MS/MS system has been developed for analysis of tRNA modifications in cell and urine samples, validating m^3^C and m^1^A as promising biomarkers for bladder cancer diagnosis, and that a panel of m^3^C, m^1^A, m^1^G, and m^2,2^G could be used as biomarkers for bladder cancer prognosis [457]. A sensitive hydrophilic interaction liquid chromatography and tandem mass spectrometry (HILIC-MS/MS) method, coupled with stable isotope dilution, was developed for precise quantification of methylated nucleosides in human urine, and this analysis revealed that the contents of m^1^G and m^5^C in urine were elevated in patients with triple-negative breast cancer (TNBC) compared to those with non-TNBC [458]. A sensitive high-performance liquid chromatography (HPLC) method has also been established for the detection of modified urinary nucleosides, including cytidine, m^2,2^G, m^2^G, m^1^G, Ψ, and m^1^A, which may serve as potential biomarkers for cancer [459].

#### tRNA modification enzymes as genetic biomarkers

Beyond direct detection of modified nucleosides, the genetic and transcriptional status of tRNA-modifying enzymes themselves holds prognostic and predictive value. For instance, SNPs in modifier genes have been linked to cancer susceptibility, such as the rs236110 C > A polymorphism in *TRMT6* which is associated with increased risk of Wilms tumor and hepatoblastoma in pediatric cohorts [355, 356]. Additionally, mutations in mitochondrial tRNA modifier genes such as *GTPBP3* and *MTO1* are linked to mitochondrial diseases and may also predispose to cancer [460, 461]. Furthermore, dysregulated expression of these enzymes is frequently observed in malignancies. Overexpression of ‘writers’ such as METTL1, NSUN2, PUS7, and ELP3 correlates with poor prognosis, advanced stage, and metastatic potential across multiple cancer types, including HCC, head and neck squamous cell carcinoma, glioblastoma, and breast cancer [35, 36, 247, 364, 365, 386, 387]. Conversely, the downregulation of tumor suppressive modifiers such as TYW2 or NSUN6 is correlated with aggressive disease [361, 405]. These findings support the potential of incorporating tRNA modifier expression signatures into multi-omics prognostic panels and further highlight their potential as therapeutic targets.

#### tRNA-derived RNAs as a new class of cancer biomarkers

tRNA-derived RNAs are increasingly recognized as stable and abundant small RNAs in biofluids, and their modification status may add an additional layer of diagnostic information. Importantly, tdRs can retain the chemical modifications of their parental tRNAs, which may influence their stability, protein-binding properties, and biological functions [55, 168, 374]. Specific tdR signatures have been associated with cancer diagnosis, progression, and therapeutic response. In terms of diagnostic potential, distinct tdR profiles can distinguish cancer patients from healthy controls. For instance, a panel of tdRs was able to discriminate breast cancer patients with high accuracy [462]. Regarding prognostic value, elevated levels of specific tdRs, such as 5′-tiRNA-Pro^TGG^ in CRC, correlate with poor survival and recurrence [463], while in myelodysplastic syndrome, dysregulated tdR signatures predict leukemic transformation [179]. Furthermore, as predictive biomarkers, tdRs can modulate therapy response. The m^7^G-modified 3'-tiRNA^Lys^_TTT_ promotes bladder cancer malignancy and may serve as a predictor of response to targeted therapies [374], and 5’tiRNA-Gly-GCC confers 5-FU resistance in CRC, suggesting its potential to guide chemotherapy selection [416]. The presence of these modifications can be inferred from sequencing data. Su et al. demonstrated that tdRs harboring m^1^A, m^1^G, or m^2,2^G modifications can be identified by characteristic mismatches during TGIRT sequencing [464]. This suggests that the modification status of tdRs, rather than merely their sequence, carries valuable biological information. However, whether tdRs undergo *de novo* modification after cleavage from full-length tRNAs remains an open question that warrants further investigation. Given their stability and disease-associated signatures, tdRs and their modifications hold promise as novel biomarkers for cancer detection and monitoring [48, 152, 465].

## Targeting tRNA modifications for cancer treatment

### Genetic therapeutics targeting tRNA modification enzymes

An innovative RNA therapeutic strategy has been devised that employs RNA interference (RNAi) to selectively target and induce the degradation of mRNA associated with tRNA regulators, obviating the necessity for an indepth understanding of structural characteristics of target proteins [29]. At present, several small interfering RNA (siRNA) drugs leveraging this technology are either available on the market or undergoing clinical trials [466]. In addition to exogenous siRNA therapeutics, screening of endogenous microRNA enables the precise targeting of tRNA regulators. For example, microRNA-214 suppresses the expression of *TRMT6/TRMT61A*, a tRNA m^1^A methyltransferase, thereby diminishing cell proliferation in cancer [168, 354, 467].

### Regulators of tRNA modification as potential anticancer drug candidates

In light of the role of tRNA modification enzymes in regulating chemotherapy and targeted therapy efficacy, they are promising drug targets for boosting cancer therapy. Of particular interest is U_34_ enzymes that are potential therapy targets for overcoming acquired resistance to targeted therapies [423], and m^7^G and m^5^C modification enzymes that might potentiate chemotherapy-induced DNA damage and apoptosis. NSUN3, catalyzing m^5^C in mt-tRNAs and mitochondrial translation of oxidative phosphorylation complex, is a potential target for inhibiting tumor metastasis [40]. Moreover, certain antibiotics, such as tigecycline and doxycycline, which specifically target translation in the mitochondria, may serve to inhibit tumor metastasis [40]. As multiple tRNA modification enzymes are deregulated in cancer, it appears feasible that inhibition of tRNA-modifying enzymes can selectively compromise cancer cells while sparing their normal counterparts, representing drug targets for cancer therapy. Substantial efforts are being directed toward the identification and development of small molecule weight inhibitors targeting tRNA-modifying enzymes [468–471]. Whilst inhibitors targeting tRNA modifications remain in experimental phase, there is growing anticipation that strategic modulation of tRNA modification will lead to novel therapeutics.

### Targeting modification-regulated tdRs in cancer

Accumulating evidence indicates that tRNA modifications play a crucial role in modulating tumorigenesis by governing the biogenesis and regulatory functions of tdRs [465]. Studies have explored the use of tdR mimetics and antisense oligonucleotides directed against tdRs for therapy purposes, these are prone to degradation by endogenous RNases in the bloodstream or removed from circulation [472]. Significant advances in drug delivery systems have the potential to improve targeting of tdRs in vivo. For instance, poly(amino esters) (PAEs)-based polymeric nanoparticles are biodegradable and exhibit pH-responsive properties [473], and has been demonstrated to deliver inhibitors of oncogenic molecule 5'tRNA-Gly-GCC, enabling precise targeting in combination with 5-FU (PAE@^5-FU^tdR-inhibitor) to CRC tissues in mouse models, whilst avoiding toxicity to distant organs [416].

### Microbial tdRs as potential therapeutic agents against cancer

Although the gut microbiota is increasingly acknowledged as critical for human health and disease, the biological activities of tdRs derived from bacteria have received scant attention in research. Recent studies have recognized tdRs that harbor specific chemical modifications, such as Gm, as a new class of bioactive constituents originating from gut microbes [406, 474]. The modified tdRs from bacteria and fungi have demonstrated cytotoxic effects against cancer cells [406, 407]. Additionally, the incorporation of base chemical modifications into tdRs can enhance their inherent properties. For instance, artificial 2′-*O*-methylation (Nm) of saliva-derived tdRs augmented their antibacterial effect against *Fusobacterium nucleatum*, a recognized causative pathogen of CRC [475]. These findings indicate tdRs as promising therapeutics against cancer.

### Artificial tRNAs for cancer therapy

**Fig. 7:**
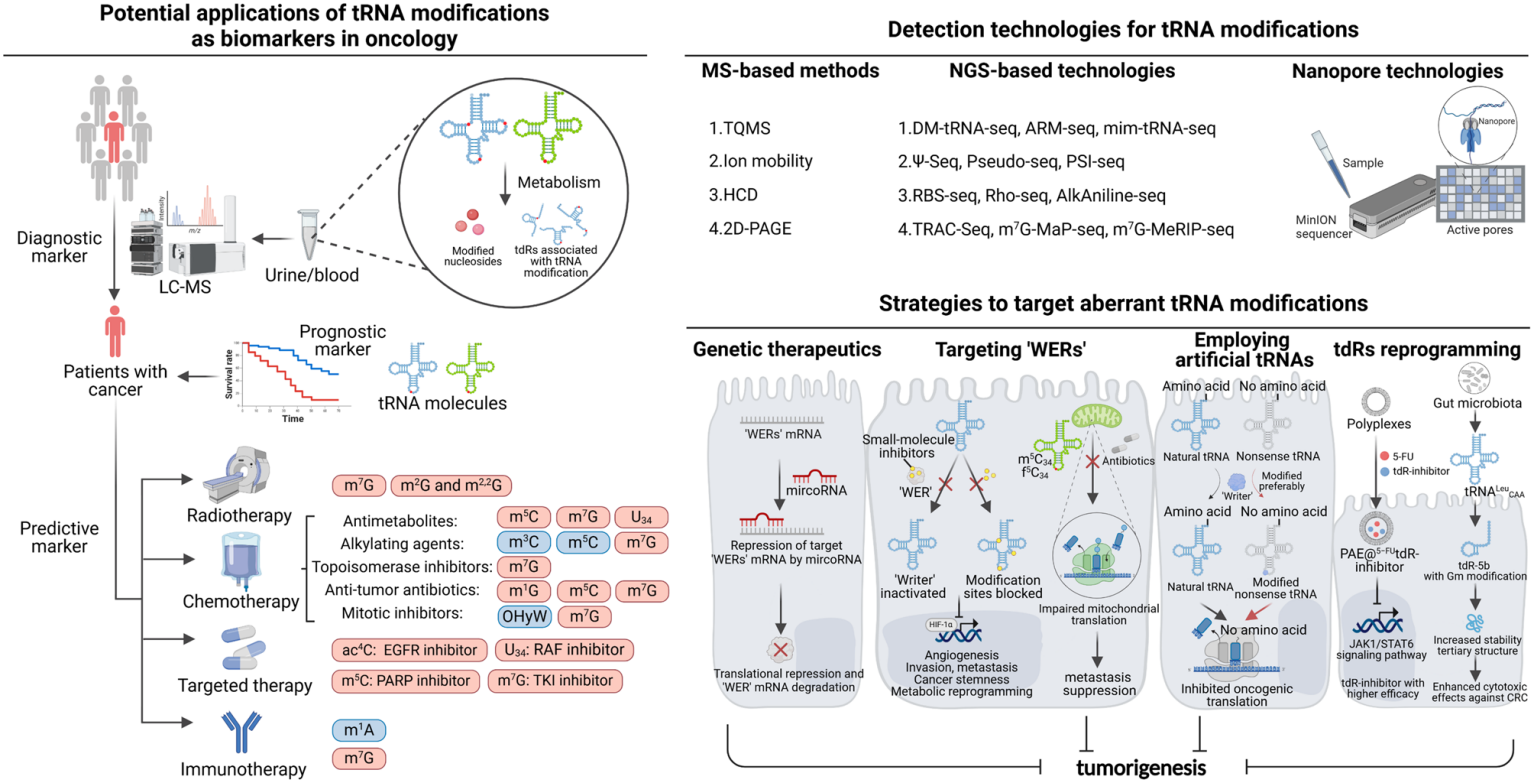
Innovative methodologies for the detection of tRNA modifications and their clinical implications as biomarkers in the prevention and treatment of cancer. Transfer RNA (tRNA) modification profiles and signatures hold promise as diagnostic biomarkers for cancer screening and detection, prognostic indicators for evaluating patient outcomes, and predictive biomarkers to screen patients likely to benefit from cancer treatments, such as radiotherapy, chemotherapy, molecularly targeted therapies, or immunotherapy. tRNA modifying enzymes identified as key determinants of response to each treatment modality are enumerated. Modifiers linked to sensitivity for the respective therapies are highlighted in blue, signifying that patients with tumors exhibiting a specific level of the modifier are more likely to experience therapeutic benefit. Conversely, the modifiers associated with resistance to the respective treatments are denoted in red, indicating that patients with abnormal intratumoral expression of these modifiers tend to have suboptimal responses. The development of new sequencing methodologies aimed at achieving more precise and comprehensive detection of diverse tRNA modifications is currently a prominent area of research interest. Existing approaches primarily encompass liquid chromatography-mass spectrometry (LC-MS)-based, next-generation sequencing (NGS)-based technologies and engineered nanopore sequencing. Data from emerging studies highlight the potential of targeting the aberrant tRNA modifications. For example, the tRNA modification process can be effectively disrupted by small molecule compounds, either by directly targeting tRNA regulators or by binding to specific modification sites on the tRNA. 2D-PAGE, two-dimensional polyacrylamide gel electrophoresis; 5-FU, 5-fluorouracil; CRC, colorectal cancer; HCD, higher-energy collisional dissociation; tdRs, tRNA-derived RNAs; TQMS, triple quadrupole mass spectrometer; ‘WERs’, ‘writers’, ‘erasers’, and ‘readers’

An additional strategy entails modulating the translation process through the application of artificially synthesized nonsense tRNA molecules [29]. Albers et al. [476] devised a novel strategy that involves transforming native tRNAs into highly efficient suppressor tRNAs (sup-tRNAs) via precise fine-tuning of their sequences to align with the physicochemical properties of the amino acids that they carry. This strategy was demonstrated to overcome premature termination codon and restore the synthesis of target proteins. In the near future, it might be possible to generate nonsense tRNA that competes with endogenous tRNAs for tRNA modification enzymes, thereby blocking undesirable tRNA modifications. Ongoing interest in aberrant tRNA modifications in cancer will likely spark new interest in artificial tRNAs research for cancer prevention and treatment (Table 4; Fig. 7).

**Table 4:** Implications of tRNA modifications in cancer: from bench to bedside

Application area	Specific strategy/ Biomarker	Key targets/modifications	Related cancer type(s)	Mechanism of action
**Diagnosis & Prognostication**	Liquid biopsy biomarkers	m^3^C, m^1^A	Bladder cancer	Non-invasive diagnosis via urine analysis
		Panel: m^3^C, m^1^A, m^1^G, m^2,2^G	Bladder cancer	Prognostication
		m^1^G, m^5^C	Triple-Negative Breast Cancer (TNBC)	Differentiation from non-TNBC via urine analysis
		Cytidine, m^2,2^G, m^2^G, m^1^G, Ψ, m^1^A	Cancer (General)	Potential universal biomarkers detected in urine
	tdR-based biomarkers	tdRs from m^1^A, m^1^G, m^2,2^G	Cancer (General)	Detection via TGIRT sequencing; potential for cancer detection and monitoring
**Therapeutic targeting**	Genetic therapeutics	siRNA/miRNA targeting modification enzymes	Cancer (General)	Degrade mRNA of tRNA regulators (e.g., miR-214 inhibits TRMT6/61A) to diminish proliferation
	Small molecule inhibitors	U_34_ enzymes	Cancer (General)	Overcome acquired resistance to targeted therapies
		m^7^G, m^5^C enzymes	Cancer (General)	Potentiate chemotherapy-induced DNA damage and apoptosis
		NSUN3 (m^5^C)	Cancer (General)	Inhibit mitochondrial translation and tumor metastasis
		Mitochondrial translation	Cancer (General)	Antibiotics (e.g., Tigecycline, Doxycycline) inhibit metastasis
	Targeting tdRs	tdR mimetics/antisense oligonucleotides	Cancer (General)	Modulate tumorigenesis; require advanced delivery systems to avoid degradation
		Nanoparticle-delivered tdR inhibitors (e.g., PAE@5-FUtdR-inhibitor)	CRC	Precisely target oncogenic tdRs (e.g., 5'tRNA-Gly-GCC) in combination therapy
	Microbial tdRs	Bacterially/fungally derived modified tdRs	Cancer (General)	Exhibit cytotoxic effects against cancer cells
		Chemically modified tdRs (e.g., 2′-*O*-methylation)	CRC	Enhance antibacterial effect against CRC-associated pathogens (e.g., *F. nucleatum*)
	Artificial tRNAs	Suppressor tRNAs (sup-tRNAs)	Cancer (General)	Readthrough premature termination codons to restore functional protein synthesis
		Synthetic nonsense tRNAs	Cancer (General)	Compete for modification enzymes to block oncogenic tRNA modifications

^*^ CRC, colorectal cancer; siRNA, small interfering RNA; TGIRT, thermostable group II reverse transcriptase; tdRs, tRNA-derived RNAs

## Conclusions and future perspectives

### General progress

The past decade has witnessed remarkable progress in elucidating the complex landscape of tRNA modifications and their functional significance in cancer biology. From early observations of dysregulated modified nucleosides in cancer patient biofluids to sophisticated mechanistic insights into how specific modifications at defined tRNA positions influence tumorigenesis, metastasis, therapy resistance, and immune evasion, the field has matured considerably. We now recognize that tRNA modifications are not merely static decorations ensuring structural integrity but are instead dynamic epitranscriptomic marks that meticulously regulate protein synthesis, produce bioactive small non-coding RNAs, and coordinate cellular responses to stress and metabolic cues.

### Knowledge gaps and limitations

Despite significant progress in elucidating the role of tRNA modifications in cancer, several critical limitations remain. First, the interplay between different modifications on the same tRNA molecule and how combinatorial modification patterns influence tRNA function, stability, and tdR biogenesis in cancer remains to be fully understood. Secondly, it is integral to decipher how tRNA modification patterns in tdRs, as the tRNA fragments may harbor unique functionalities distinct from their parent tRNAs. Moreover, the potential biological functions of tdRs originating from bacteria to host tumor cells have been infrequently explored, suggesting that future research should focus more on these microbe-derived therapeutics. Investigation of microbial tdRs might unlock a new class of RNA-based therapeutics against cancer.

### Translational outlook: biomarkers and therapeutics

The clinical translation of tRNA modification research is an area of active investigation, with several promising avenues emerging. First, tRNA modification regulators themselves are emerging as prognostic tissue biomarkers, with overexpression of METTL1, NSUN2, and PUS7 linked to poor patient outcomes. Second, urinary modified nucleosides (e.g., m^1^A, m^3^C) and circulating tdRs are advancing as non-invasive diagnostic and predictive biomarkers. tdRs are particularly promising for liquid biopsy applications. Their stability and cancer-specific expression patterns position them as potential tools for early detection, prognosis, and prediction of therapeutic response (e.g., to 5-FU or targeted therapies). Third, the therapeutic targeting of tRNA modification enzymes represents an emerging frontier. Realizing this potential will require dissecting their catalytic and non-catalytic oncogenic functions, as well as the incorporation of modification-based biomarkers into clinical trial design to enable patient stratification and personalized treatment strategies.

## Data Availability

No datasets were generated or analysed during the current study.

## Abbreviations

3'UTR: 3' untranslated regions
5-FU: 5-Fluorouracil
aaRSs: Aminoacyl-tRNA synthetases
ac^4^C: *N*^4^-acetylcytidine
acp^3^U: 3-(3-amino-3-carboxypropyl)uridine
ADAT2: Adenosine deaminase tRNA specific 2
AGO: Argonaute
AKT: Protein kinase B
ALKBH1: AlkB homolog 1
ANG: Angiogenin
ANXA2: Annexin A2
ASL: Anticodon stem-loop
BCDIN3D: Bicoid interacting 3 domain containing RNA methyltransferase
CDK10: Cyclin-dependent kinase 10
CDK5RAP1: Cdk5 regulatory subunit-associated protein 1
Cm: 2′-*O*-methylcytidine
cmnm^5^s^2^U: 5-carboxymethylaminomethyl-2-thiouridine
cmnm^5^U: 5-carboxymethylaminomethyluridine
CREB3L2: CAMP-responsive element-binding protein 3 like 2
CRC: Colorectal cancer
CTU1/CTU2: Cytosolic thiouridylase subunits 1/2
Cyt c: Cytochrome c
cyto-tRNAs: Cytosolic tRNAs
D: Dihydrouridine
DDR: DNA damage repair
DNMT2: DNA methyltransferase 2
DRS: Direct RNA sequencing
DUS: Dihydrouridine synthase enzymes
DTWD1: DTW Domain Containing 1
ECM: Extracellular matrix
eIF4G: Eukaryotic translation initiation factor 4G
EGFR: Epidermal growth factor receptor
ELP1-6: Elongator complex proteins 1-6
ERS: Endoplasmic reticulum stress
FTO: Fat mass and obesity-associated protein
FTSJ1: FtsJ RNA Methyl-transferase Homolog 1
galQ: Galactosyl-queuosine
GBM: Glioblastoma
Gm: 2′-*O*-methylguanosine
GTPBP3: GTP binding protein 3
GWAS: Genome-wide association study
HCC: Hepatocellular carcinoma
HER2: Human epidermal growth factor receptor 2
HIF-1: Hypoxia-inducible factor-1
HILIC-MS/MS: Hydrophilic interaction liquid chromatography and tandem mass spectrometry
hm^5^C: 5-hydroxymethylcytidine
hm^5^Cm: 2′-*O*-methyl-5-hydroxymethylcytidine
HPLC: High-performance liquid chromatography
HSPCs: Hematopoietic stem and progenitor cells
I: Inosine
i^6^A: *N*^6^-isopentenyladenosine
IFN: Interferon
KEOPS: Kinase, endopeptidase and other proteins of small size
LAGE3: L antigen family member 3
LC-MS/MS: Liquid chromatography-tandem mass spectrometry
LOXL2: Lysyl oxidase-like 2
m^1^A: 1-methyladenosine
manQ: Mannosyl-queuosine
MBTPS1: membrane-bound transcription factor peptidase, site 1
m^3^C: 3-methylcytidine
m^5^C: 5-methylcytidine
mchm^5^U: 5-(carboxyhydroxymethyl)uridine methyl ester
mcm^5^s^2^U: 5-methoxycarbonylmethyl-2-thiouridine
mcm^5^U: 5-methoxycarbonylmethyluridine
mcm^5^Um: 5-methoxycarbonylmethyl-2′-*O*-methyluridine
MDS: Myelodysplastic syndrome
MDSCs: Myeloid-derived suppressor cells
METTL: Methyltransferase-like
m^1^G: 1-methylguanosine
m^2^G: *N*^2^-methylguanosine
m^2,2^G: *N*^2^,*N*^2^-dimethylguanosine
m^7^G: 7-methylguanosine
m^1^I: 1-methylinosine
mpG: 5′-methylphosphoguanosine
MS: Mass spectrometry
MSC: Multi-synthetase complex
ms^2^i^6^A: 2-methylthio-*N*^6^-isopentenyladenosine
ms^2^t^6^A: 2-methylthio-*N*^6^-threonylcarbamoyladenosine
m^6^t^6^A: *N*^6^-methyl-*N*^6^-threonylcarbamoyladenosine
MTO1: Mitochondrial translation optimization 1 protein
mTORC1: Mammalian target of rapamycin complex 1
mt-tRNAs: Mitochondrial tRNAs
m^5^U: 5-methyluridine
m^5^Um: 2′-*O*-methyl-5-methyluridine
NAT10: *N*-acetyltransferase 10
NGS: Next-generation sequencing
Nm: 2′-*O*-methylation
NSUN: NOL1/NOP2/Sun
OCR: Oxygen consumption rate
OHyW: Hydroxywybutosine
OSGEP: O-sialoglycoprotein endopeptidase
OSGEPL1: O-sialoglycoprotein endopeptidase like 1
o_2_yW: Peroxywybutosine
PARP: Poly(ADP-ribose) polymerase
PCa: Prostate cancer
PES: Pyrimidine-enriched sequences
PI3K: Phosphatidylinositol 3-kinase
PIWI: P-element induced wimpy testis
PPARδ: Peroxisome proliferator-activated receptor delta
PUS: Pseudouridine synthase
Q: Queuosine
q: Queuine
QTRT1: Queuine tRNA-ribosyltransferase catalytic subunit 1
RBPs: RNA-binding proteins
RNAi: RNA interference
RNAPIII: RNA polymerase III
RNase: Ribonuclease
ROS: Reactive oxygen species
RPTOR: Regulatory-associated protein of mTOR complex 1
RTD: Rapid tRNA decay
SAM: S-adenosylmethionine
siRNA: Small interfering RNA
SNORD97: Small nucleolar RNA, C/D box 97
SNP: Single nucleotide polymorphism
SOX9: SRY-Box transcription factor 9
sup-tRNAs: Suppressor tRNAs
t^6^A: *N*^6^-threonylcarbamoyladenosine
tdRs: tRNA-derived RNAs
TFH: T follicular helper
TFIIIB: Transcription factor IIIB
TFIIIC: Transcription factor IIIC
TGF-β2: Transforming growth factor β2
Thg1: tRNA His guanylyltransferase
THUMPD1: Thiouridylase methyltransferase and pseudouridine synthase domain-containing protein 1
tiRNAs: tRNA-derived stress-induced fragments
TICs: Tumor-initiating cells
TME: Tumor microenvironment
TNBC: Triple-negative breast cancer
TP53RK: Tumor protein p53 regulating kinase
TPRKB: TP53RK binding protein
tRFs: tRNA-derived fragments
TRUB1: TruB pseudouridine synthase family member 1
tsRNAs: tRNA-derived small RNAs
TYK2: Tyrosine kinase 2
TYW1: tRNA Wybutosine-Synthesizing Protein 1
U: Uridine
Um: 2′-*O*-methyluridine
Urm1: Ubiquitin-related modifier 1
WDR4: WD Repeat Domain 4
YBX1: Y-box binding protein 1
YRDC: yrdC N6-threonylcarbamoyltransferase domain containing
Yes1: Yes proto-oncogene 1
τm^5^U: 5-taurinomethyluridine
τm^5^s^2^U: 5-taurinomethyl-2-thiouridine
Ψ: Pseudouridine
Ψm: 2′-*O*-methylpseudouridine
